# Virtual Reality Interventions for Older Adults With Mild Cognitive Impairment: Systematic Review and Meta-Analysis of Randomized Controlled Trials

**DOI:** 10.2196/59195

**Published:** 2025-01-10

**Authors:** Qin Yang, Liuxin Zhang, Fangyuan Chang, Hongyi Yang, Bin Chen, Zhao Liu

**Affiliations:** 1 School of Design Shanghai Jiao Tong University Shanghai China

**Keywords:** Alzheimer disease, virtual reality, VR, mild cognitive impairment, meta-analysis, health care, cognitive function, memory, attention, executive function, older adults

## Abstract

**Background:**

Alzheimer disease is incurable, but it is possible to intervene and slow down the progression of dementia during periods of mild cognitive impairment (MCI) through virtual reality (VR) technology.

**Objective:**

This study aimed to analyze the effects of VR interventions on older adults with MCI. The examined outcomes include cognitive abilities, mood, quality of life, and physical fitness, including general cognitive function, memory performance, attention and information processing speed, executive function, language proficiency, visuospatial abilities, depression, daily mobility of individuals, muscle performance, and gait and balance.

**Methods:**

A total of 4 web-based databases (Web of Science, PubMed, Embase, and Ovid) were searched up to December 30, 2023, for randomized controlled trials assessing the self-reported outcomes of VR-based technology on cognition, mood, quality of life, and physical fitness in older adults (aged ≥55 years) with MCI. Two reviewers independently screened the search results and reference lists of the identified papers and related reviews. Data on the intervention components and delivery and behavioral change techniques used were extracted. A meta-analysis, risk-of-bias sensitivity analysis, and subgroup analysis were performed where appropriate to explore potential moderators. The Grading of Recommendations, Assessment, Development, and Evaluations (GRADE) approach was used to assess the quality of evidence.

**Results:**

This review analyzed 18 studies involving 722 older adults with MCI. VR was delivered through different immersion levels with VR cognitive training, VR physical training, or VR cognitive-motor dual-task training. VR interventions showed significant improvements in memory (standardized mean difference [SMD] 0.2, 95% CI 0.02-0.38), attention and information processing speed (SMD 0.25, 95% CI 0.06-0.45), and executive function (SMD 0.22, 95% CI 0.02-0.42). VR without therapist involvement improved memory as well as attention and information processing speed. VR cognitive training also resulted in significant improvements in attention and information processing speed in older adults with MCI (SMD 0.31, 95% CI 0.05-0.58). In addition, immersive VR had a significant impact on improving attention and information processing speed (SMD 0.25; 95% CI 0.01-0.50) and executive function (SMD 0.25; 95% CI 0.00-0.50). However, the effects of the intervention were very small in terms of general cognitive function, language proficiency, visuospatial abilities, depression, daily living ability, muscle performance, and gait and balance. Quality of evidence varied, with moderate ratings for certain cognitive functions and low ratings for others, based on the GRADE approach.

**Conclusions:**

VR interventions can improve memory, attention and information processing speed, and executive function in older adults with MCI. The quality of evidence is moderate to low, and further research is needed to confirm these findings and explore additional health-related outcomes.

## Introduction

Dementia is a medical condition characterized by a notable decrease in cognitive abilities, which disrupts individuals’ ability to perform occupational, familial, or social responsibilities [1] and is currently the seventh leading cause of death worldwide [2]. As the worldwide occurrence of Alzheimer disease (AD) rises, the economic burden on society will also increase significantly, with greater disease severity being linked to higher expenses [3,4]. Mild cognitive impairment (MCI) is a transitional stage of cognitive performance that occurs between normal aging and dementia [5]. Treatments can be implemented to slow down the advancement of dementia during the preclinical phase [6]. Conventional nonpharmacologic lifestyle interventions, including moderate-intensity physical activity and autonomic training, encounter challenges such as inadequate adherence, exorbitant treatment expenses, and inequities in health care accessibility [7,8]. Since there are few medications or dietary therapy that can improve cognitive function or slow MCI progression, nonpharmacological treatments have received attention [9,10].

In nonpharmacological interventions, VR has received a lot of attention. It helps the user to create a real sense of presence and immersion in the virtual world by using multiple sensory stimuli (visual, auditory, tactile, and olfactory) [11,12]. This capability has the potential to augment participants’ focus and boost the efficacy of training sessions. For patients with MCI, VR has shown potential in enhancing cognitive capacity and motor function [13-15]. VR technology has mostly been used for evaluating and diagnosing the extent of cognitive impairment, as well as for cognitive training and testing [16]. In the assessment of the degree of cognitive impairment, VR-based virtual supermarkets, virtual fire evacuation drills, and voice interaction based on VR and wearable devices have been used to detect and distinguish between older adults with MCI and healthy older adults [15,17,18]. These methods have also been used to differentiate between persons with MCI and those with dementia [19,20], which help clinicians to detect cognitive impairment at an earlier stage compared with current diagnostic models that address different cognitive deficits. Furthermore, in terms of cognitive testing and cognitive training, a growing number of studies, such as the Virtual Environmental Grocery Store [21] and the Virtual Supermarket [22], have discovered that patients’ performance in VR tasks is closely related to their performance in traditional neuropsychological tests. This demonstrates the reliability of VR-based assessment tests compared with traditional neuropsychological tests [23-25]. Furthermore, it has the potential to enhance many cognitive capacities such as general cognitive ability, memory, attention, and executive function [26-28]. VR technology can enhance noncognitive aspects of capability or condition in individuals with MCI. For instance, older adults with MCI may experience a reduction in anxiety by engaging with virtual environments such as virtual nature or tourist attractions [29]. Other studies also indicated that VR-based cognitive training (cooking or wayfinding) could effectively enhance instrumental activities of daily living (IADL) in individuals with MCI and AD [30].

Several trials have investigated the impact of VR on older adults with MCI, but the findings have been inconclusive. For example, studies by Baldimtsi et al [31] and Park et al [32] revealed a significant effect of VR on general cognitive abilities. However, the findings from Park et al [33] showed that a 12-week, culture-based VR training program did not improve general cognitive abilities and did not show significant differences in scores on the Mini-Mental Status Examination (MMSE). Also, it is challenging to make definitive conclusions about the effectiveness of interventions because of variations in the content of the interventions and the way they interact with VR. In a study conducted by Yang et al [28], daily life-based VR training games (making juice, shooting crows, finding the number of fireworks, and memorizing objects in the house) were found to positively affect general cognitive performance. However, in a study by Delbroek et al [34], which involved a combination of VR cognitive and motor training for balance, weight-bearing, memory, attention, and dual-tasking, no significant differences in general cognitive performance were observed.

Now, only 1 comprehensive assessment has examined the effects of VR on the cognitive abilities of older adults with MCI [13]. Nevertheless, it solely concentrated on the efficacy of executive function and failed to provide a thorough examination of cognitive capabilities. A comprehensive assessment found that a VR-based neuropsychological intervention was helpful in enhancing cognitive performance in individuals diagnosed with MCI [35]. However, this review did not address the different VR interventions due to the inclusion of only 1 specific intervention based on VR technology and the lack of analysis of multiple VR technology–based interventions. Currently, there is no systematic review of the VR field, and limited knowledge exists regarding the most effective type or combination of VR solutions for improving cognitive capacities, emotional performance, daily functioning, and physical fitness in older individuals with MCI. A broad overview of the evidence through a more nuanced categorization of VR content, professionals, and system-level VR solutions is required. Therefore, this review aims to assess the effects of VR technology–based interventions on older adults, specifically those with MCI. The outcomes include cognition, mood, quality of life, and physical fitness. Also, it is important to identify the most effective combination of the most effective components (eg, VR intervention content, provider, and level of immersion) for use in clinical and managerial responses to the evidence, as well as policy decisions.

## Methods

### Systematic Literature Review

Guidance published in PRISMA (Preferred Reporting Items for Systematic Reviews and Meta-Analyses) [36] and the Cochrane Handbook of Systematic Reviews [37] was adhered to. The a priori protocol for the review is published in the International Prospective Register of Systematic Reviews (PROSPERO; CRD42024503488).

### Search Strategy

Studies were identified by searching web-based databases with support and consultation provided by institutional librarians. Searches included Web of Science, PubMed, Embase, and Ovid using both Medical Subject Headings and free-text keywords relating to MCI, VR interventions, and outcomes. To include studies reflecting the latest advancements in VR technology and methodologies, the focus was on literature published from January 2013 to December 2023. The year 2013 was chosen to ensure consistency in the technological sophistication and usability standards of VR interventions, as VR technology and its applications in cognitive rehabilitation have rapidly evolved over the past decade. One of the two pre-2013 papers were excluded due to unspecified levels of cognitive impairment, which could have affected the identification of cognitive deficits [38]. The other paper was excluded because cognitive deficits were due to conditions such as stroke or cerebral infarction, to maintain sample specificity [39]. Therefore, the outcomes from January 2013 to December 2023 are listed below. Search strategies for the first 3 databases above and dates on which searches were conducted are listed in Multimedia Appendix 1. Searches of the reference list of the previous review papers and included studies were also conducted.

### Eligibility Criteria

#### Type of Studies

Randomized controlled trials (RCTs) of any design, including parallel-group, crossover, and cluster RCTs published in the English language were included in this review.

#### Population, Intervention, Comparison, Outcome Framework

This study uses the PICO (Population, Intervention, Comparison, Outcome) framework to structure the research question and guide the systematic review and meta-analysis. The PICO approach is a well-established method for formulating research questions and conducting systematic reviews in health care research [40].

#### Participants

Studies concerning older adults (aged ≥55 years) with a confirmed diagnosis of MCI by neurologic examination or neuropsychological assessment were included. The age cutoff of 55 years and older was chosen because individuals in this age group are eligible for certain age-related benefits and programs in various contexts, such as early retirement plans, senior discounts, and health interventions targeting midlife transitions. This makes it a practical cutoff for researching populations that can access interventions aimed at promoting active aging. No restrictions were applied to the study design. Studies with healthy older adults; persons with schizophrenia, depression, or Parkinson disease; and participants who did not specify the level of cognitive deficits were excluded.

#### Intervention

The intervention criterion considered the use of virtual environments, virtual interactive means, and VR components, including VR-based physical training, VR-based cognitive training, VR-based cognitive-motor dual-task training, face-to-face cognitive VR rehabilitation system, and VR programs.

#### Control Condition

Studies with any type of control group were included (inactive controls include educational programs or no intervention; active controls include traditional rehabilitation or any other type of physical activity, physical-cognitive co-training, or video games without VR components).

#### Outcomes

We included any psychometric, physical functioning measure that was reasonably unidimensional or multidimensional and the relevant subscales associated with the outcomes in Textbox 1.

**Measurement scales related to the outcomes.**

**Primary Outcomes**

- General cognitive function: the Mini-Mental State Examination, the Montreal Cognitive Assessment, Korean version of the Modified Mental State Examination-Dementia Screening Test, the Cognitive Abilities Screening Instrument, Subcategory VST of CNT 4.0, Montreal Cognitive Assessment and GP Cognitive Assessment (dementia screening tool), and Loewenstein Occupational Therapy Cognitive Assessment – Aging
- Memory performance [41]: the Digit Span Test; Rey Auditory Verbal Learning Test; Wechsler Memory Scale, Third Edition; Multifactorial Memory Questionnaire; Chinese Verbal Learning Test; 20-item version of the Everyday Memory Questionnaire (range: 20-180); and Seoul Verbal Learning Test
- Attention and information processing speed: Trajectory Making Test A and B, Symbol Digit Substitution Test, Attention Matrix Test, and Digit Span Test
- Executive function: Korean version of the Executive Function Performance Test, Symbol Digit Substitution Test, Trajectory Making Test B, Stroop Color and Word Test, Executive Interview 25, and Frontal Assessment Battery

**Secondary Outcomes**

- Language proficiency: Word Fluency Test (category and letter fluency), Verbal Fluency (phonological fluency; semantic fluency), Battery for Analysis of Aphasic Deficits, and Korean version of the Boston Naming Test
- Visuospatial abilities: Rey-Osterrieth Complex Figure Test, Clock Drawing Test, and Weschsler Adult Intelligence Scale Revised Block Design Test
- Depression: 30-item Geriatric Depression Scale, 15-item Geriatric Depression Scale, and Multidimensional Observation Scale for Elderly Subjects
- The daily mobility of individuals: the Lawton Instrumental Activities of Daily Living scale, the Quality of Life in Alzheimer's Disease scale, and the Multidimensional Observation Scale for Elderly Subjects
- Gait and balance: Gait Speed Test, Timed Up and Go Test, Instrumented Timed Up and Go Test, limit of stability (LOS) values (a subcategory of RM Ingénierie's BIORescue system), and Six Minute Walk Test
- Muscle performance: Hand Gauge Measurement, Arm Flexion Test, and Lower Body Strength using the 30-second Sit-Stand Test

### Exclusion Criteria

The exclusion criteria were as follows:

1. Not RCTs: Studies that were not RCTs were excluded.
2. Participants’ age ≤55 years: Studies where the participants’ average age was 65 years or younger were excluded.
3. Not participants with MCI: Studies that did not include participants diagnosed with MCI were excluded.
4. Cognitive impairment caused by other conditions: Studies where cognitive impairment was attributed to other medical conditions, such as stroke, cerebral infarction, traumatic brain injury, or other neurological disorders, were excluded. This ensures that the cognitive impairment under study is specifically related to MCI and not secondary to other health issues.
5. No specific identification of cognitive impairment: Studies where VR was not used in the intervention group, or VR was used in the control group, were excluded.
6. Inappropriate use of VR: Studies where VR was not used in the intervention group, or VR was used in the control group, were excluded.

### Minimum Studies for Analysis

Analyses were conducted only if there were at least 3 studies available for each specific outcome measure. This criterion ensures the reliability and validity of the meta-analytic results.

### Study Selection and Data Extraction

Two reviewers (QY and ZL) independently screened the abstracts and full text of the search results. Any disagreements were resolved through discussion. Data were extracted by 2 researchers (HY and LZ) and cross-checked by a third researcher (FC). Data items included participant characteristics, intervention device, intervention means, expertise or guidance, duration of intervention, adherence, and fidelity.

### Meta-Analysis

We used RevMan (version 5.3; Cochrane Collaboration) for all analyses. Outcome data were expressed as standardized mean difference (SMD) and were pooled in a pairwise fixed-effects model stratified based on the outcome and type of digital medium. We used the mean (SD) of within-group change from baseline to calculate the SMDs. Per Cohen [42], SMDs <0.2 were classified as small, those between 0.2 and 0.8 were classified as medium, and those >0.8 were classified as large. The SMD was converted to the percentage change in the outcome measure by multiplying the SMD with the SD of the control group of the sufficiently powered study (ie, the trial with the largest sample size).

A sensitivity analysis was conducted for the risk-of-bias assessment. Subgroup analyses were conducted to assess the effect of VR on the different immersion effects used, the different intervention components, and the presence or absence of therapist involvement in the intervention. Heterogeneity across pooled studies was examined using the chi-squared test and *I*^2^ statistics. We did not conduct additional exploratory analyses to explore potential moderators because of an insufficient number of studies. The common study characteristics tables were supplemented by additional summary tables including the risk-of-bias assessment; effect estimates; Grading of Recommendations, Assessment, Development, and Evaluations (GRADE) analysis; and behavior change taxonomy groupings.

### Dealing With Missing Data

RevMan calculator was used to estimate missing SDs based on test statistics reported in 2 studies. In addition, sensitivity analyses were conducted to assess the robustness of the results when missing data were imputed. Imputation methods were based on the assumption that data were missing at random.

### Risk-of-Bias and GRADE Assessment

Risk of bias was assessed using the risk of bias tool of the Cochrane Handbook for Systematic Reviews [43]. Quality of evidence for outcomes was assessed according to the 5 GRADE domains, including study limitation (risk of bias), inconsistency, indirectness, imprecision, and publication bias [44,45].

## Results

### Study Selection and Characteristics

**Figure 1:**
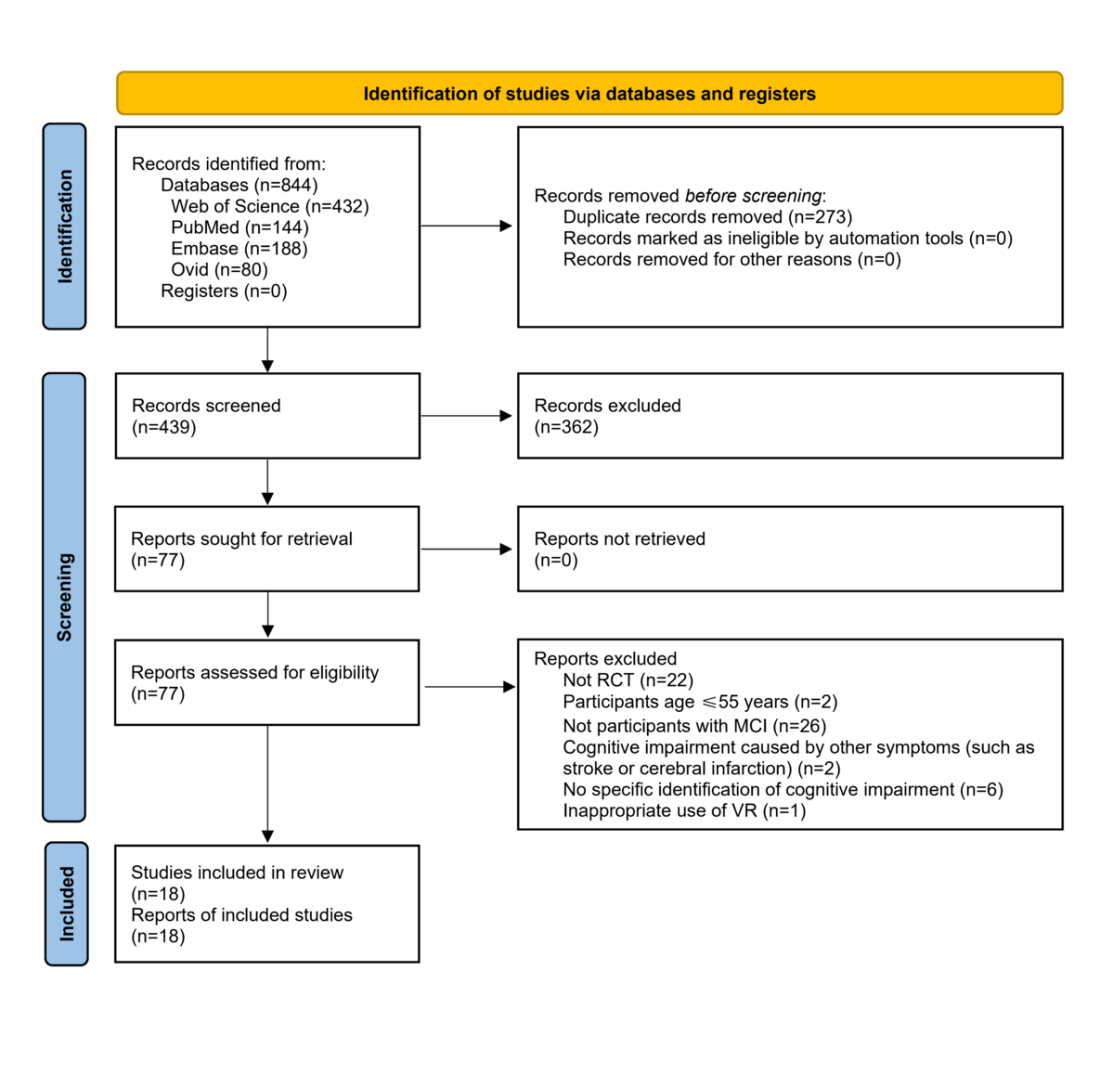
PRISMA (Preferred Reporting Items for Systematic Reviews and Meta-Analyses) flow diagram. MCI: mild cognitive impairment; RCT: randomized controlled trial; VR: virtual reality.

A total of 571 titles and abstracts were screened after excluding duplicates, of which 494 records did not meet the inclusion criteria (Figure 1). The full text of 78 potentially eligible records was read, including 18 studies published between 2017-2023. These studies included 722 (range 17-68) older adults with MCI. In total, 18 studies were RCTs [26-28,31-34,46-56]. Specific study characteristics and participant demographics are summarized in Multimedia Appendix 2.

### Participants

In total, 18 studies were included, with a total of 8 studies conducted in South Korea; 2 in Hong Kong, China; 2 in Taipei, China; and 6 in mainland China, Greece, Brazil, France, Turkey, and Japan.

The number of participants in the studies ranged from 17 to 68. The median age was 73.15 (IQR 66.07-87.5) years. Most participants in the studies were female (median 63.6%, IQR 16.7%-86.7%).

Nine studies reported participants’ years of education, with a median age of 8.56 (IQR 7.20-9.90) years. Ten studies reported participants’ baseline MMES scores, with a median score of 26.28 (IQR 25.30-27.4).

Detailed information on the characteristics of the included studies and the demographic data of the study participants are provided in the Multimedia Appendix 2.

### Intervention Groups

Nine studies used VR headsets as the medium for VR intervention gear. Among these, 2 studies incorporated additional components, while 7 studies used stand-alone screens or projections. Furthermore, 6 studies were equipped with other components. One study used desktop computers, while 2 studies used tablets. Regarding control and perception, 10 studies used controllers while 4 studies used wearable devices for VR interventions. In terms of cognitive interventions, 15 studies were conducted, with 3 studies focusing on a single cognitive intervention modality and 12 studies using an integrated cognitive intervention system. In 9 of the studies conducted, 2 studies focused solely on motor interventions without involving cognitive interventions, whereas the remaining 7 studies included both motor and cognitive interventions. A total of 14 studies incorporated a gamification element, 9 studies included a feedback component, and 7 studies implemented a progressive difficulty level. Professional input and support were included in all of them. Specifically, 4 studies involved specialists who provided their expertise during the development of the intervention content, while 12 studies had a therapist involved in the intervention throughout its duration. Two studies did not provide relevant descriptions. Regarding the duration of the VR intervention, 3 studies had an intervention period of 4 weeks, 3 studies had an intervention period of 6 weeks, 5 studies had an intervention period of 8 weeks, 6 studies had an intervention period of 12 weeks, and 1 study had an intervention period of 24 weeks. For detailed information on the intervention implementation mechanisms, refer to Multimedia Appendix 2.

### Comparison

VR-based interventions were compared with treatment as usual in 2 studies, combined physical and cognitive training group in 3 studies, conventional cognitive rehabilitation group in 3 studies, attention group (health education) in 2 studies, physical exercise group in 2 studies, computer and video games in 1 study, and no intervention in 5 studies.

### Risk of Bias

**Figure 2:**
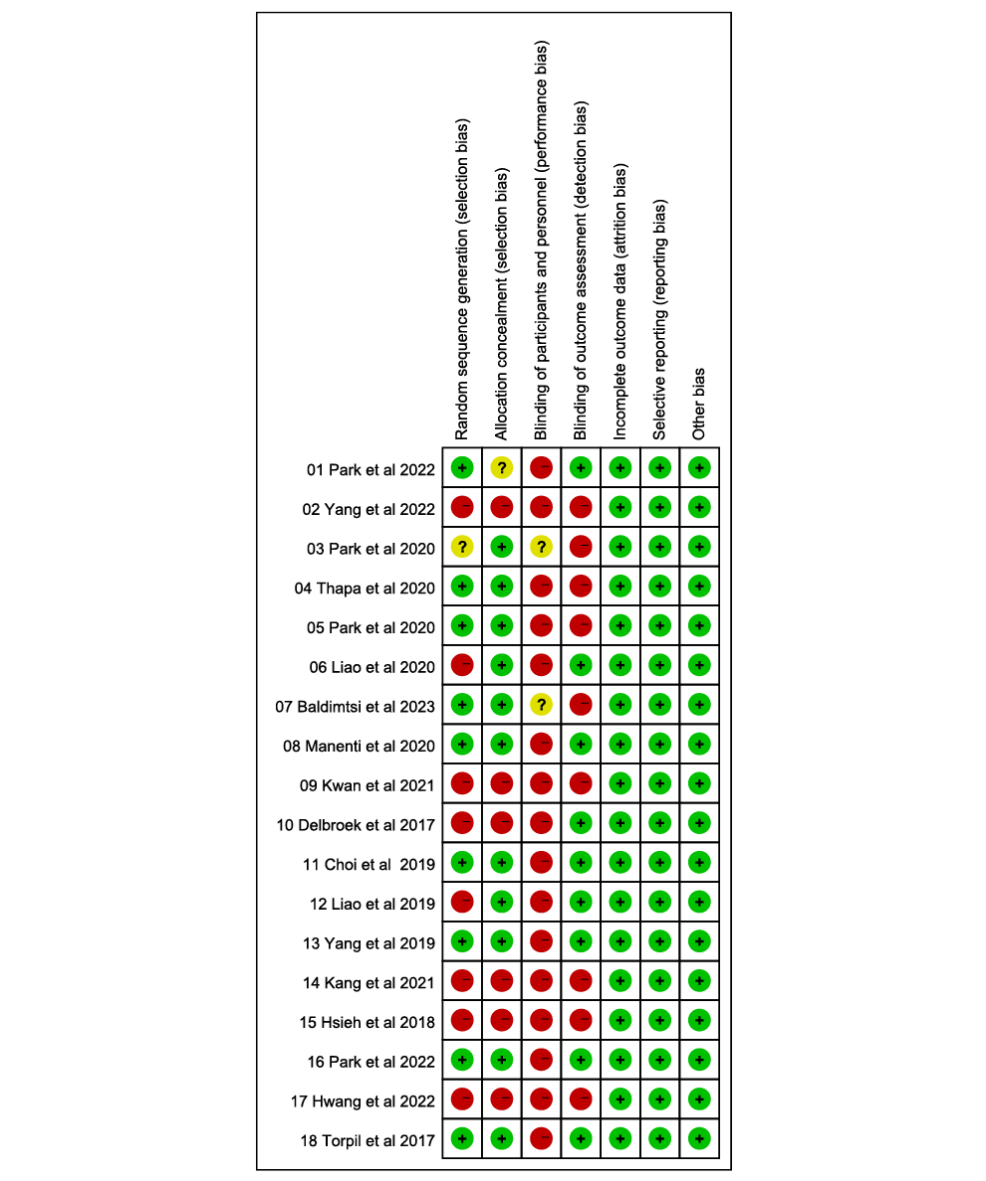
Summary of the risk-of-bias assessment.

The risk of bias was unclear in 1 study for random sequence generation, 1 study for allocation hiding, and 2 studies for performance bias. A high risk of bias was observed in 8 studies for random sequence generation, 6 studies for allocation concealment, 16 studies for performance bias, and 9 studies for detection bias (Figure 2 [26-28,31-34,46-56]). Due to the nature of the interventions and patient-reported outcome instruments, both detection bias and performance bias were rated high in most studies.

### Effects of Interventions

The main comparative results of the study with GRADE ratings are summarized in Multimedia Appendix 3.

### Main Outcomes

#### Primary Outcomes

##### General Cognitive Ability

**Figure 3:**
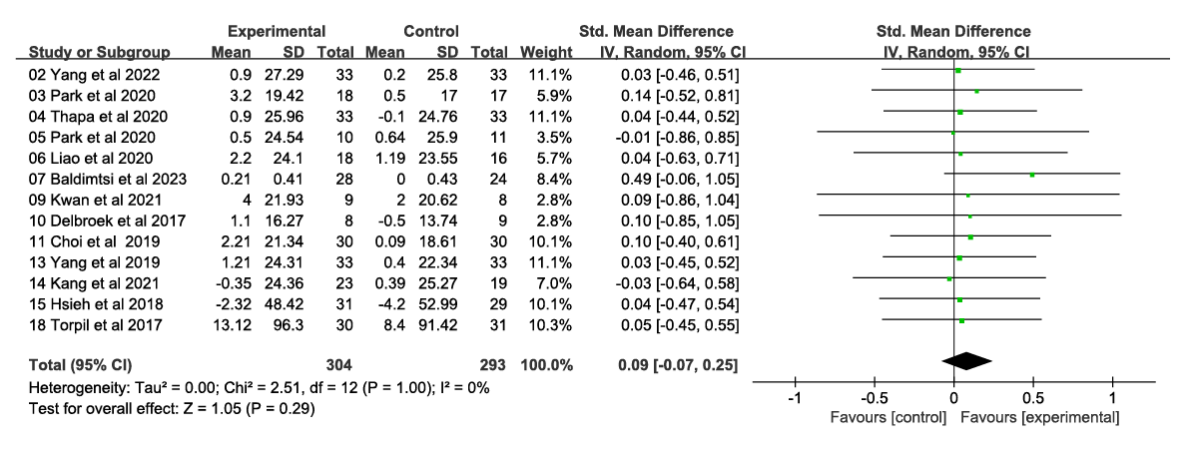
Forest plot of general cognitive ability: comparison of general cognitive ability improvement at postintervention time points based on virtual reality intervention versus conventional treatment or no intervention control group.

A total of 13 studies involving 597 participants reported on the effects of VR technology-based interventions on general cognitive function performance at postintervention time points (range 4-24 weeks) compared with conventional control conditions (Figure 3 [26-28,31-34,47,49,50,52-54]). In summary, the intervention had a small impact on general cognitive function, but this effect was not statistically significant (SMD 0.09, 95% CI −0.07 to 0.25); there was also no significant heterogeneity (*χ*^2^_12_=2.51, *P*>.99; *I*²=0%). Using the GRADE approach, the quality of evidence was rated moderate because of the high risk of bias in most studies (ie, study limitations).

##### Performance and Memory

**Figure 4:**
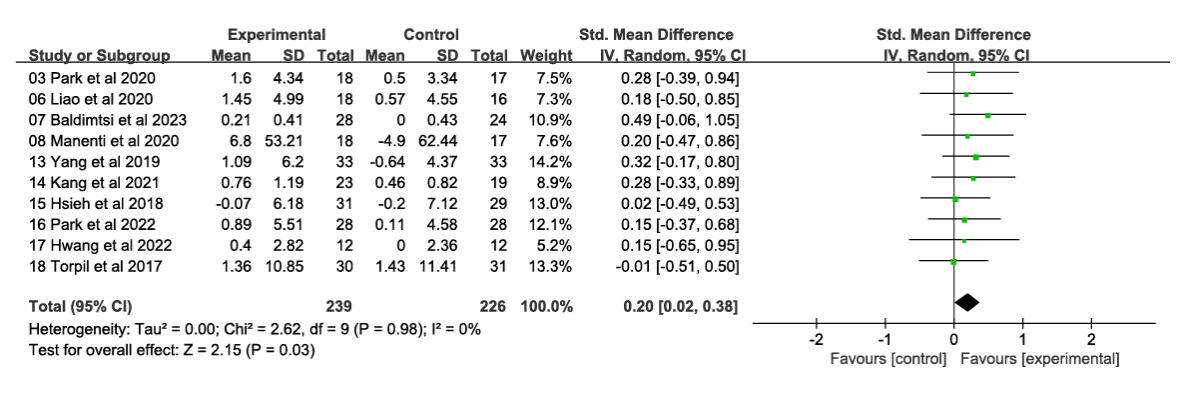
Forest plot of performance and memory: comparison of performance and memory improvement at postintervention time points based on virtual reality intervention versus conventional treatment or no intervention control group.

Ten studies, with a total of 465 participants, examined the impact of a VR technology–based intervention on performance and memory (Figure 4 [27,31,32,47,48,52-56]). The studies measured these effects at various time points after the intervention, ranging from 4 to 24 weeks. The VR technology–based intervention was compared with a traditional control condition. The overall effect sizes showed statistically significant and moderately sized impacts on performance and memory (SMD 0.20, 95% CI 0.02-0.38). There was some heterogeneity in the results (*χ*^2^_9_=2.62, *P*=.08; *I*²=0%). The general cognitive function evidence was deemed moderate due to the high risk of bias in most studies (ie, study limitations).

##### Attention and Information Processing Speed

**Figure 5:**
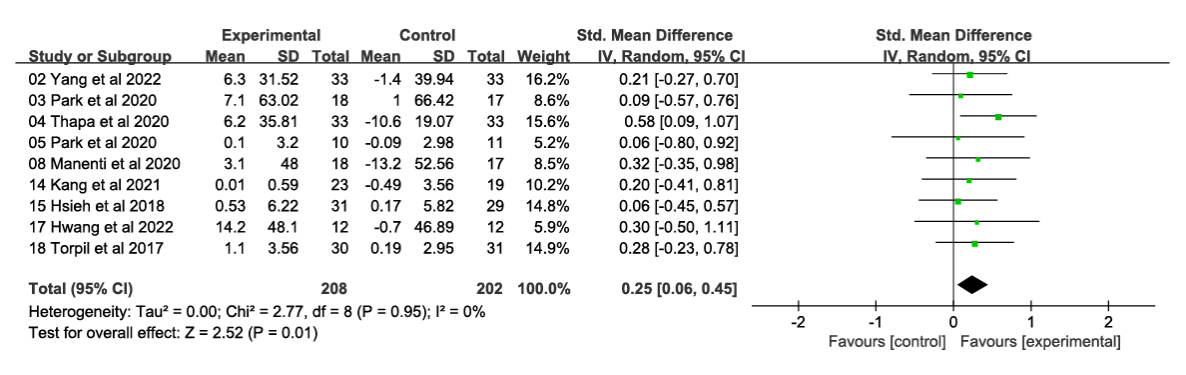
Forest plot of attention and information processing speed: comparison of attention and information processing speed improvement at postintervention time points based on virtual reality intervention versus conventional treatment or no intervention control group.

A total of 9 studies involving 410 participants reported on the effects of a VR technology–based intervention on attention and information processing speed at postintervention time points (range 4-24 weeks) compared with a conventional control condition (Figure 5 [26-28,32,33,48,53,54,56]). Overall effect sizes indicated significant and moderate effects on attention and information processing speed (SMD 0.25, 95% CI 0.06-0.45); heterogeneity (*χ*^2^_8_=2.77, *P*=.95; *I*²=0%). The quality of evidence for attention and information processing speed was rated as moderate due to the high risk of bias (ie, study limitations).

##### Executive Function

**Figure 6:**
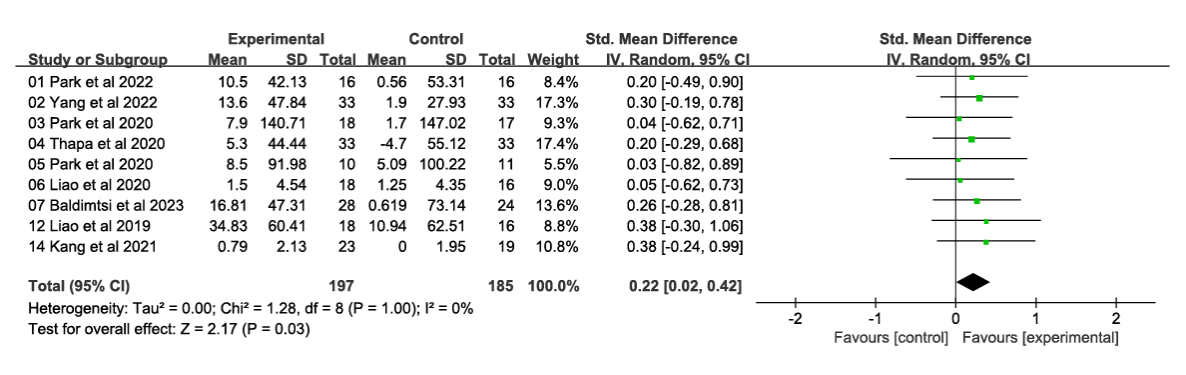
Forest plot of executive function: comparison of executive function improvement at postintervention time points based on virtual reality intervention versus conventional treatment or no intervention control group.

In total, 9 studies, with a total of 382 participants, examined the impact of VR technology–based intervention on executive function (Figure 6 [26,28,31-33,46,47,51,53]). These studies measured the effects at postintervention time points, which ranged from 4 to 12 weeks, and compared them with traditional control conditions. The intervention showed substantial and moderate effects on executive function (SMD 0.22, 95% CI 0.02-0.42). There was no heterogeneity among the studies (*χ*^2^_8_=1.28, *P*>.99; *I*²=0%). The evidence on implementation capacity was assessed as intermediate in quality due to the high risk of bias (ie, study limitations).

#### Secondary Outcomes

No significant findings were seen for any of the secondary outcomes (Multimedia Appendix 4). The study found that there were no significant effects on language proficiency (SMD 0.21, 95% CI −0.20 to 0.61) and the quality of evidence was rated as very low. However, there were significant effects on visuospatial abilities, depression, and the daily mobility of Individuals, with effect sizes of 0.24 (95% CI −0.09 to 0.57), 0.06 (95% CI −0.29 to 0.42), and 0.10 (95% CI −0.23 to 0.43), respectively. The quality of evidence for these effects was rated as low. The study found that the impact sizes for gait and balance were statistically negligible, with an SMD of 0.05 and a 95% CI ranging from −0.17 to 0.27. The quality of evidence was considered moderate.

#### Subgroup Analysis

##### Therapist Involvement

###### Performance and Memory

**Figure 7:**
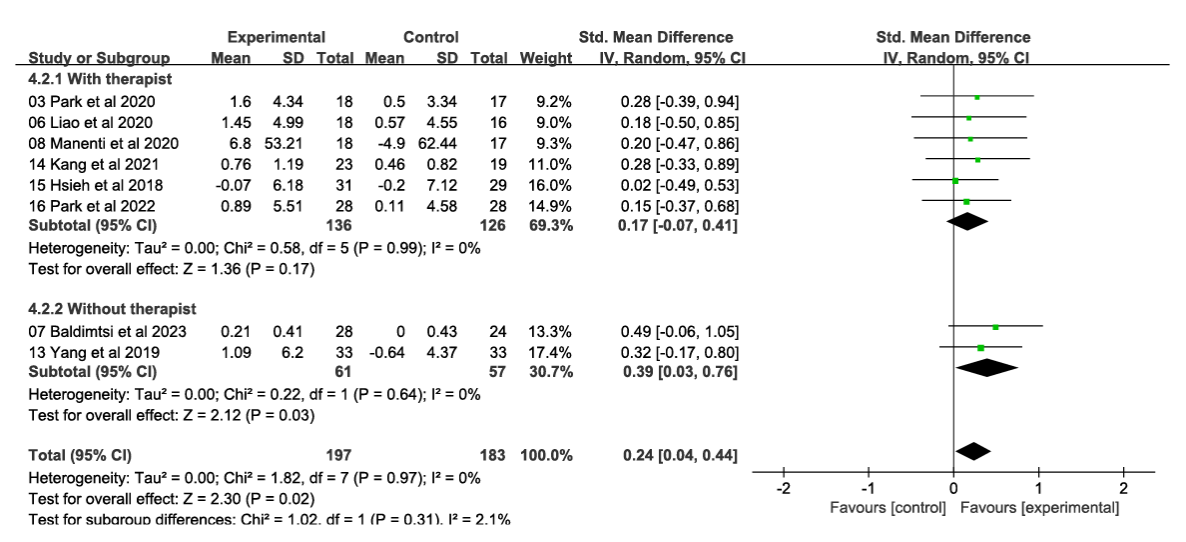
Forest plot of subgroups with and without therapist participation (performance and memory): comparison of performance and memory at postintervention time points based on therapist involvement or no therapist involvement.

In total, 2 studies involving 118 participants found that interventions to improve performance and memory had significant and moderate effects (SMD 0.39, 95% CI 0.03-0.76). These studies did not involve therapist involvement (Figure 7 [31,32,47,48,52-55]). There was no heterogeneity in the results (*χ*^2^=0.22, *P*=.64; *I*²=0%). On the other hand, 6 studies involving 262 participants did involve therapist involvement but did not find significant effects favoring performance and memory interventions (SMD 0.17, 95% CI −0.07 to 0.41). There was also no heterogeneity in these results (*χ*^2^_5_=0.58, *P*=.99; *I*²=0%). Nevertheless, there was no substantial distinction observed between the 2 groups (*χ*^2^_1_=1.02, *P*=.31; *I*²=2.1%).

###### Attention and Information Processing Speed

**Figure 8:**
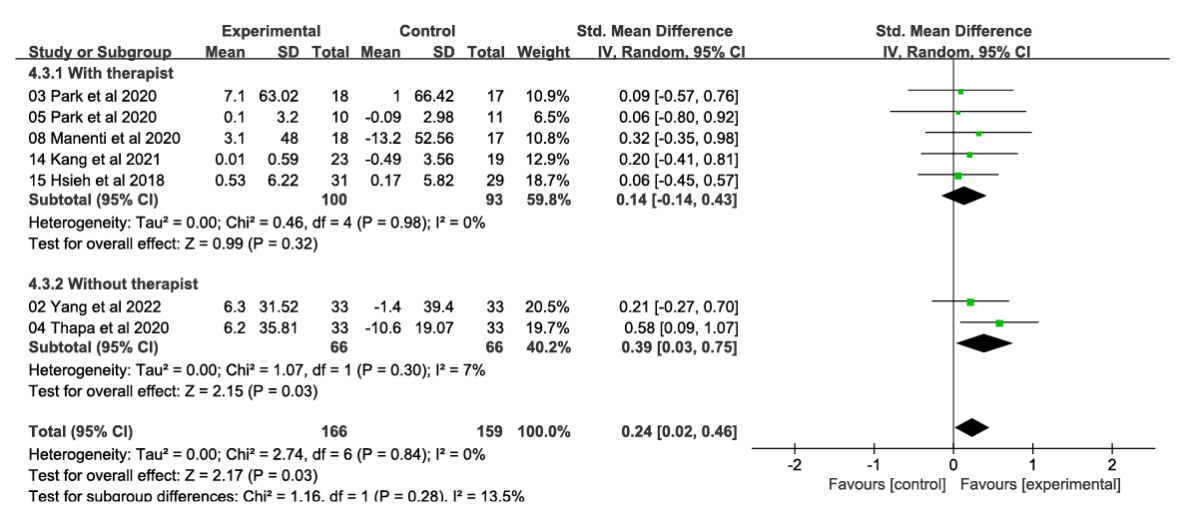
Forest plot of subgroups with and without therapist participation (attention and information processing speed): comparison of attention and information processing speed at postintervention time points based on therapist involvement or no therapist involvement.

Two studies involving 132 participants uncovered no therapist involvement results (Figure 8 [26,28,32,33,48,53,54]), with significant and moderate effects observed in favor of interventions to improve attention and information processing speed (SMD 0.39, 95% CI 0.03-0.75); there was no heterogeneity (*χ*^2^_1_=1.07, *P*=.30; *I*²=7%). In contrast, 5 studies involving 193 participants uncovered results of therapist involvement but did not observe a significant effect in favor of the attention and information processing speed intervention (SMD 0.14, 95% CI –0.14 to 0.43); there was no heterogeneity (*χ*^2^_4_=0.46, *P*=.98; *I*²=0%). However, the difference between the 2 groups was not significant (*χ*^2^_1_=1.16, *P*=.28; *I*²=13.5%).

##### Content of the Intervention

###### Attention and Information Processing Speed

**Figure 9:**
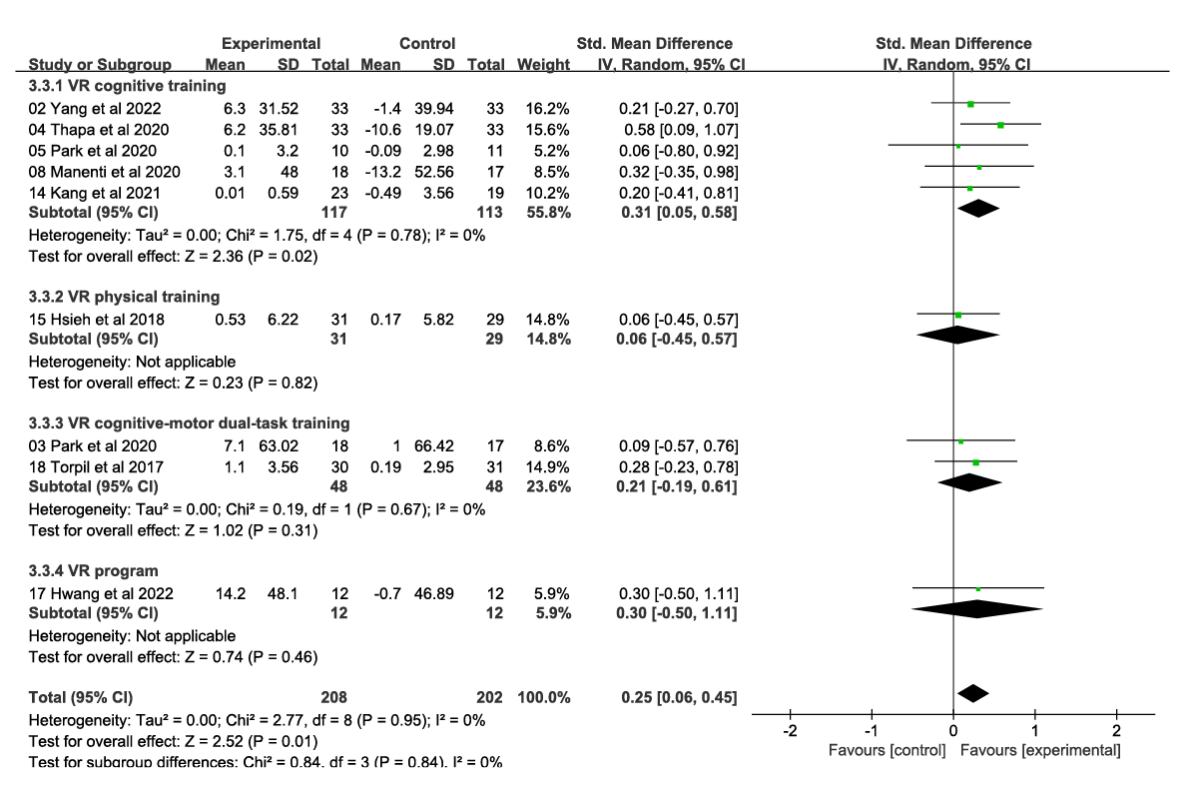
Forest plot of subgroups by intervention content (attention and information processing speed): comparison of virtual reality cognitive training, virtual reality physical training, virtual reality cognitive-motor dual-task training, and virtual reality program in improving attention and information processing speed at postintervention time points.

VR cognitive training (5 studies, 230 participants) produced significant and moderate effects in improving attention and information processing speed (SMD 0.31, 95% CI 0.05-0.58; *χ*^2^_4_=1.75, *P*=.78; *I*²=0%; Figure 9 [26-28,32,33,48,53,54,56]). The other 3 intervention components—VR physical training (1 study, 60 participants; SMD 0.06, 95% CI −0.45 to 0.57), VR cognitive-motor dual-task training (2 studies, 96 participants; SMD 0.21, 95% CI −0.19 to 0.61; *χ*^2^_1_=0.19, *P*=.67; *I*²=0%), and VR program (1 study, 24 participants; SMD 0.30, 95% CI −0.50 to 1.11)—did not have a significant effect on attention and information processing speed. The difference between the 4 groups was not significant (*χ*^2^_3_=0.84, *P*=.84; *I*²=0%).

##### Level of Immersion

###### Attention and Information Processing Speed

**Figure 10:**
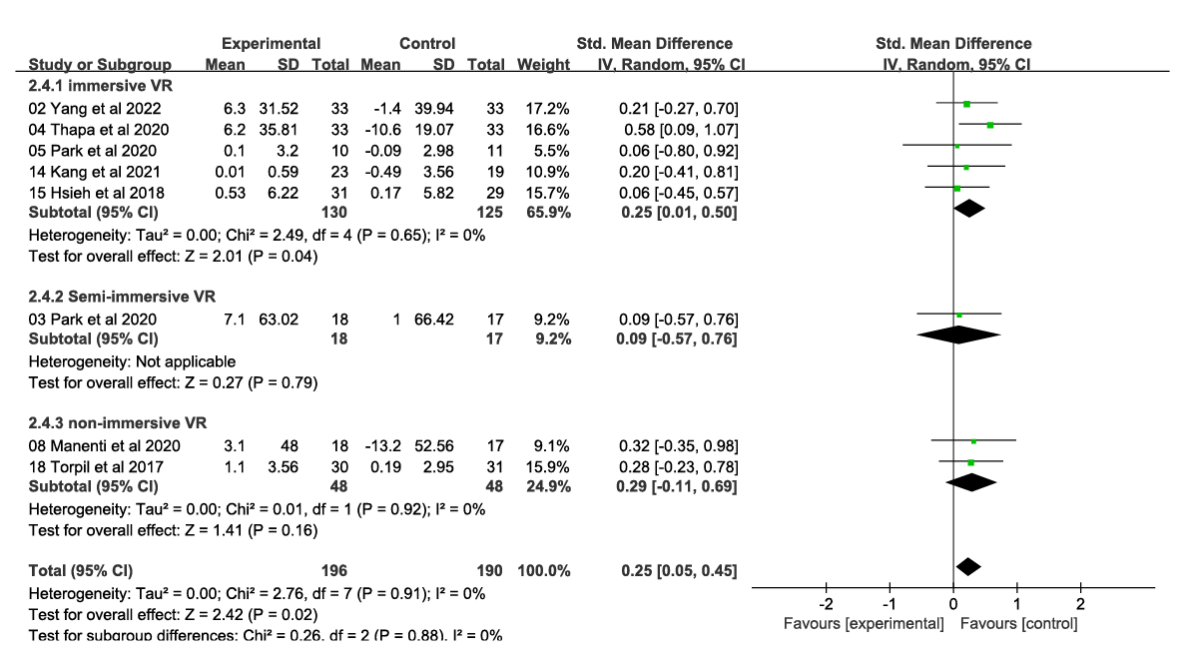
Forest plot of subgroups by immersion level (attention and information processing speed): Immersive, semi-immersive, nonimmersive, and combined immersive and semi-immersive immersion levels improved attention and information processing speed at postintervention.

Subgroup analyses based on level of immersion showed that immersive VR interventions (5 studies, 255 participants) produced significant and moderate effects on improving attention and information processing speed (SMD 0.25, 95% CI 0.01-0.50; *χ*^2^_4_=2.49, *P*=.65; *I*²=0%; Figure 10 [26-28,32,33,48,53,54]). Semi-immersive VR interventions (1 study, 35 participants; SMD 0.09, 95% CI −0.57 to 0.76) and nonimmersive VR interventions (2 studies, 96 participants; SMD 0.29, 95% CI −0.11 to 0.69; *χ*^2^_1_=0.01, *P*=.92; *I*²=0%) had no significant effects on attention and information processing speed. The difference between the 3 groups was not significant (*χ*^2^_2_=0.26, *P*=.88; *I*²=0%).

#### Executive Function

**Figure 11:**
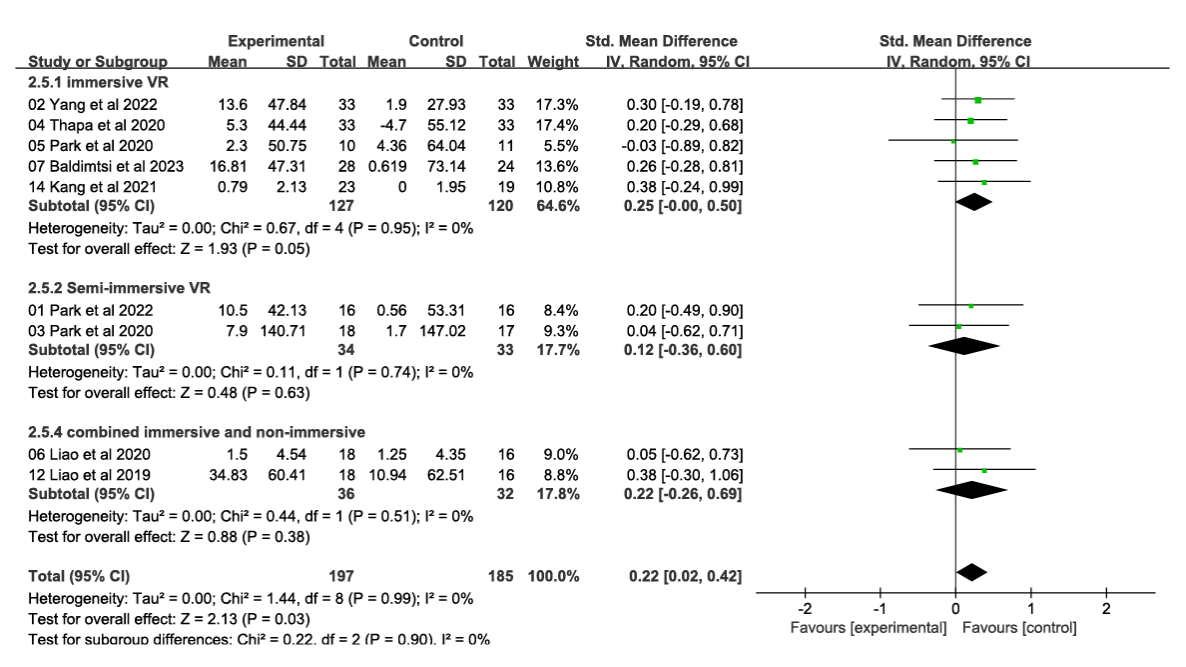
Forest plot of subgroups by immersion level (executive ability): comparison of immersion, semi-immersive, nonimmersive, and combined immersive and semi-immersive immersion levels in improving executive ability at postintervention time points.

Subgroup analyses based on level of immersion showed that immersive VR interventions (5 studies, 247 participants) produced significant and moderate effects in improving executive function (SMD 0.25, 95% CI 0.00-0.50; *χ*^2^_4_=0.67, *P*=.95; *I*²=0%; Figure 11 [26,28,31-33,46,47,51,53]). Semi-immersive VR interventions (2 studies, 67 participants; SMD 0.09, 95% CI −0.57 to 0.76; *χ*^2^_1_=0.11, *P*=.74; *I*²=0%) as well as combined immersive and nonimmersive VR interventions (2 studies, 68 participants; SMD 0.22; 95% CI −0.26 to 0.69; *χ*^2^_1_=0.44, *P*=.74; *I*²=0%) had no significant effect in executive function. The difference between the 3 groups was not significant (*χ*^2^_2_=0.22, *P*=.90; *I*²=0%).

#### Risk-of-Bias Sensitivity Analysis

There was little evidence of funnel plot asymmetry in the intervention effects for general cognitive function, performance and memory, attention and speed of information processing, and executive function (Multimedia Appendix 5). In addition, excluding studies with no or unclear random allocation, inadequate concealment of treatment allocation, or incomplete outcome data, the effect sizes were as follows: improved general cognitive function (SMD 0.12, 95% CI −0.11 to 0.35); improved performance and memory (SMD 0.30, 95% CI 0.02-0.57); improved attention and information processing speed (SMD 0.41, 95% CI 0.05-0.77); and improved executive function (SMD 0.14, 95% CI −0.16 to 0.44). All the RCTs included in this analysis are parallel RCTs.

## Discussion

### Principal Findings

The findings of this review indicate that using VR as an intervention resulted in significant improvements in performance and memory (SMD 0.20, 95% CI 0.02-0.38), speed of attention and information processing (SMD 0.25, 95% CI 0.06-0.45), and executive function (SMD 0.22, 95% CI 0.02-0.42). Our study did not discover any notable disparities in general cognitive function and any secondary outcomes. However, the findings should be interpreted with caution as there is a risk of bias in the included studies, and therefore, the overall quality of the evidence is moderate and low.

This review examined how VR impacts performance and memory in older adults with MCI (SMD 0.20, 95% CI 0.02-0.38). Of the 10 included studies that assessed performance and memory, 9 showed positive changes. The results were similar to the study by Zhu et al [14]. This may be due to the fact that memory traces formed under the immersion of a virtual experience have richer content and a more complex associative network, and are part of an extensive autobiographical associative network [57,58]. A study showed that participants in the VR condition mostly relied on the mechanism of autobiographical memory, namely vivid recall, for retrieving memory task items. In contrast, participants in the 2D personal computer-game condition primarily relied on familiarity for retrieval [59]. VR offers advantages such as improved attitudes and heightened desire for training, in comparison with conventional motor or cognitive therapies [60]. Furthermore, VR has the ability to stimulate mirror neurons and improve individual empowerment and self-confidence by integrating perception, cognition, and action [61]. The findings of our study indicate that the use of VR intervention has a notable impact on both performance and memory. However, it is important to note that achieving this good effect may necessitate more comprehensive training during the VR intervention.

The effects of VR on the population with MCI in terms of attention and information processing speed were investigated in 9 included studies, all of which showed positive changes (SMD 0.25, 95% CI 0.06-0.45). The findings align with the research conducted by Yu et al [13], Gomez-Caceres et al [35], and Ren et al [62]. Previous studies demonstrated significant activation of the cuneate lobe, middle occipital gyrus, and regions involved in emotional processing (eg, the insula) during VR experiences [63]. In contrast, the right insula may be activated by a combination of attentional and response control demands that play a role in processing sensory stimuli related to the current target [64]. During VR attention training, engagement in visual attention tasks is associated with a number of significantly enhanced activation clusters, and stereoscopic presentation in VR may facilitate attentional engagement by reducing the metabolic costs of intermediate stages of visual processing [65].

The study examined the impact of VR on the executive function of individuals with MCI (SMD 0.22, 95% CI 0.02-0.42). All 9 of the included studies demonstrated beneficial changes. However, the results conflict with Yan et al [15]. This may be due to the fact that they focused only on VR in the form of combined cognitive and physical interventions, whereas combined interventions may lead to excessive stress and a reduction in the cognitive benefits of cognitive training or physical activity in combined interventions [66]. Most of the VR interventions in our study were goal-oriented tasks with IADL [14,35]. Executive function plays a dominant role in a person’s ability to adapt to situations that arise in daily life [67,68]. Therefore, active experience-dependent plasticity triggered by task-oriented training leads to neurological changes in the brain and improves daily life performance [69]. Although this reference concerns poststroke patients, it is hypothesized that similar mechanisms of plasticity might be relevant for individuals with MCI engaged in VR-based cognitive training, based on the shared principles of neuroplasticity. Using IADL as a goal-directed task may improve the effectiveness of executive function interventions. Therefore, future research should consider this variable when developing intervention strategies.

### Subgroup Analysis

The results of subgroup analyses show that the VR intervention without therapist involvement had better results in terms of improving performance and memory as well as in terms of attention and speed of information processing compared with the VR intervention with therapist involvement. The results conflict with the study of Manenti et al [48]. This may be due to the bias in this study [48], where the experimental and control groups were not equal in terms of intervention time, intervention measures, and the number of interventions was small. The lack of therapist guidance may have motivated participants to use cognitive strategies more actively, resulting in more effective processing of information and improved performance. Autonomous learning experiences can enhance individuals’ cognitive processes, leading to deeper thinking and better integration of knowledge, ultimately resulting in more enduring memory effects. This aligns with the beneficial impact of self-directed learning on the strengthening of memory retention as observed in previous research [70-72]. Furthermore, a study conducted by Plancher et al [73] has noted that the advantages of active conditioning mostly rely on procedural abilities and the consequences of personal engagement in the encoding process. In terms of attention and information processing speed, users in contexts without therapist involvement were more likely to focus on the task itself, reducing the influence of external incentives and cognitive distractions, resulting in more significant cognitive outcomes. In contexts with therapist involvement, users may be more susceptible to social comparisons and evaluations. The therapist’s guidance and feedback may become part of the cognitive load and add additional psychological stress [74,75], which may have a negative impact on attention and information processing speed. In addition, the presence of a therapist introduces extrinsic motivation that may cause users to focus more on the therapist’s expectations and feedback than on the task itself, which in turn affects the effectiveness of task performance [76]. It is important to note that this does not imply that the trainer is redundant in all contexts, but rather emphasizes that VR interventions without trainer intervention may be more conducive to achieving significant results in terms of performance and memory, as well as in terms of attention and speed of information processing, in specific contexts. Future research could further explore the interactive effects of different task types and individual differences on the presence or absence of trainer involvement to more fully understand the mechanisms behind this finding. Also, future research should be designed with more attention to user privacy issues to provide users with a stronger sense of security.

The results of the subgroup analysis regarding the content of the VR intervention indicate that VR cognitive training produces significant effects in improving attention and information processing speed compared with VR physical training, VR cognitive-motor dual-task training, and VR programs. This may be due to the fact that VR physical training and VR programs require lower intrinsic cognitive load, and VR cognitive training requires a higher cognitive load. The participants’ cognitive systems are more actively engaged, thus stimulating more active cognitive processes [77]. In addition, exercise requires cognitive provision (engagement of attention, memory, and executive function) to fulfill its function [78]. Compared with single-tasking, dual-tasking is performed with higher brain activation in the prefrontal cortex and requires more cognitive resources [79]. Due to the limited capacity of the cognitive system, excessive cognitive load may affect learning and execution of the task [80]. VR cognitive training focuses on a specific cognitive task, whereas VR cognitive motor dual-task training may have introduced more distractions or loads that affected cognitive performance. Therefore, future VR intervention content should carefully consider the relationship between cognitive tasks and motor share, and should be designed to be more suitable for MCI patients.

Our study also find that immersive VR produced significant effects in terms of improving attention and information processing speed and in terms of executive function compared with semi-immersive VR, nonimmersive VR, and combined immersive and semi-immersive VR intervention situations. The results are similar to the study by Zhu et al [14] but conflicted with the study by Yu et al [13], which stated that semi-immersive VR was superior to fully immersive VR and nonimmersive VR in promoting cognitive flexibility. However, in recent years, VR technology has evolved and the use of immersive VR technology has been used to provide a more profound and holistic sensory experience through the creation of immersive virtual environments with more extensive sensory encounter [81]. The increased level of immersion in VR can promote interaction between the patient and the task, thus improving ecological validity [82]. Cognitive training in VR environments can enhance cognitive function in reality by facilitating the transfer of cognitive talents from games to real-life situations, thanks to cognitive function and brain plasticity [69,83]. However, the results should be interpreted with caution, as the included studies are at risk of bias, imprecise, and inconsistent, and therefore, the overall quality of the evidence is low or very low.

### Limitations

This review has a number of limitations. First, only studies published in English were considered. Second, it is difficult to blind interventions and outcome assessments; as a result, all studies endure performance and detection bias. In addition, some of the most robust studies were at high risk of bias, reducing the overall quality of the evidence. Third, the sample sizes of most studies were small, limiting the statistical power and generalizability of the results, and secondary outcomes such as language proficiency, depression, and the daily mobility of Individuals were only reported in 4 studies. Fourth, defining thresholds of dysfunction remains a difficult task, and the included studies differed in inconsistency in their inclusion criteria for participants with MCI. Fifth, the age cutoff was set at 55 years and older, with the awareness that the definition of older adults varies across cultures, as some reviews include participants aged ≥40 years [84] and ≥65 years [13]. Given that many researchers have begun advocating against using age as the sole criterion for classifying older adults, future reviews could consider relaxing the inclusion criterion on age and include studies that investigate interventions for active aging. Finally, most studies did not take into account factors that can modulate participants’ cognitive functioning, mood, the daily mobility of Individuals, and physical fitness (eg, cognitive reserve, diet, sleep, and physical and cognitive activities outside of the intervention). However, our findings can still provide compelling insights into the future of VR intervention design for older adults with MCI.

### Conclusion

This review of 18 RCTs evaluates the effects of VR interventions on cognitive abilities, emotional functioning, daily mobility, and physical fitness in older adults with MCI. We found notable improvements in memory performance, attention, information processing speed, and executive function. VR training, particularly without therapist involvement, led to enhancements in memory, attention, and processing speed. These findings suggest that VR interventions can be an effective tool for cognitive rehabilitation in older adults with MCI, offering a practical approach to support their cognitive health and potentially delay the progression of dementia. Further research is needed to compare VR programs and validate these findings, considering patient diversity, intervention duration, and measurement accuracy.

## Appendix

Multimedia Appendix 1 Search strategy.

Multimedia Appendix 2 Characteristics and demographics of included studies.

Multimedia Appendix 3 Summary of findings and Grading of Recommendations, Assessment, Development, and Evaluations ratings for the main comparisons.

Multimedia Appendix 4 Forest plots of secondary outcomes.

Multimedia Appendix 5 Funnel plots of review comparisons.

Multimedia Appendix 6 PRISMA checklist.

## Abbreviations

AD: Alzheimer disease
GRADE: Grading of Recommendations, Assessment, Development, and Evaluations
IADL: instrumental activities of daily living
LOS: limit of stability
MCI: mild cognitive impairment
PICO: Population, Intervention, Comparison, Outcome
PRISMA: Preferred Reporting Items for Systematic Reviews and Meta-Analyses
PROSPERO: International Prospective Register of Systematic Reviews
RCT: randomized controlled trial
SMD: standardized mean difference
VR: virtual reality
