# Supplementary material for: Virtual Reality Interventions for Older Adults With Mild Cognitive Impairment: Systematic Review and Meta-Analysis of Randomized Controlled Trials
Source: J Med Internet Res. 2025 Jan 10;27:e59195. doi: 10.2196/59195 (PMC11759915; doi:10.2196/59195)
Supplement: Multimedia Appendix 1 [file jmir_v27i1e59195_app1.pdf]

Appendix 1: Search Strategy

Web of Science

Date searched: 29 December 2023

#	Search	Results
1	("virtual reality" OR "VR" OR "VE" OR " virtual environment" OR "virtual game") AND ("older adults" OR "geriatric" OR "elderly") AND ("dementia" OR "Alzheimers" OR " Amentia" OR " Amentias" OR " Dementias " OR "MCI" OR "cognitive impairment" OR " Mild Cognitive Impairment " OR " Cognitive Impairment, Mild" OR " Cognitive Dysfunction" OR " Dysfunction, Cognitive" OR " Cognitive Impairments" OR " Cognitive Disorder" OR " Decline, Cognitive") AND ("cognitive training" OR "cognition" OR "depression" OR "PTSD" OR "anxiety" OR "mood") (Topic)	516
2	#1 and 2023 or 2022 or 2021 or 2020 or 2019 or 2018 or 2017 or 2016 or 2015 or 2014 or 2013 (Publication Years)	456
3	#2 and English (Languages)	423

PubMed

Date searched: 29 December 2023

#	Searches	Results
1	("virtual reality" OR "VR" OR "VE" OR " virtual environment" OR "virtual game") AND ("older adults" OR "geriatric" OR "elderly") AND ("dementia" OR "Alzheimers" OR " Amentia" OR " Amentias" OR " Dementias " OR "MCI" OR "cognitive impairment" OR " Mild Cognitive Impairment " OR " Cognitive Impairment, Mild" OR " Cognitive Dysfunction" OR " Dysfunction, Cognitive" OR " Cognitive Impairments" OR " Cognitive Disorder" OR " Decline, Cognitive") AND ("cognitive training" OR "cognition" OR "depression" OR "PTSD" OR "anxiety" OR "mood")	169
2	#1 AND ((2013:2023[pdat])	145
3	#2 AND (english[Filter])	144

EMBASE

Date searched: 29 December 2023

#	Searches	Results
1	'virtual reality' OR 'vr' OR 've' OR 'virtual environment' OR 'virtual game'	181,553

Sensitivity: Internal

2	'older adults' OR 'geriatric' OR 'elderly'	1,004,325
3	'dementia' OR 'alzheimers' OR 'amentia' OR 'amentias' OR 'dementias' OR 'mci' OR 'cognitive impairment' OR 'mild cognitive impairment' OR 'cognitive impairment, mild' OR 'cognitive dysfunction' OR 'dysfunction, cognitive' OR 'cognitive impairments' OR 'cognitive disorder' OR 'decline, cognitive'	549,943
4	'cognitive training' OR 'cognition' OR 'depression' OR 'ptsd' OR 'anxiety' OR 'mood'	1,632,442
5	#1 AND #2 AND #3 AND #4	321
6	#5 AND (2013:py OR 2014:py OR 2015:py OR 2016:py OR 2017:py OR 2018:py OR 2019:py OR 2020:py OR 2021:py OR 2022:py OR 2023:py)	256
7	#6 AND ([aged]/lim OR [middle aged]/lim OR [very elderly]/lim) AND [english]/lim	188

Sensitivity: Internal
