# Supplementary material for: Virtual Reality Interventions for Older Adults With Mild Cognitive Impairment: Systematic Review and Meta-Analysis of Randomized Controlled Trials
Source: J Med Internet Res. 2025 Jan 10;27:e59195. doi: 10.2196/59195 (PMC11759915; doi:10.2196/59195)
Supplement: Multimedia Appendix 2 [file jmir_v27i1e59195_app2.pdf]

JMIR Publications
Advancing Digital Health & Open Science
www.jmirpublications.com

Publication Agreement

V2023.2 - Effective March 2023

The manuscript with the title **Virtual Reality Interventions for Older Adults with Mild Cognitive Impairment: a Systematic Review and Meta-analysis of Randomized Controlled Trials** authored by Qin Yang, Liuxin Zhang, Fangyuan Chang, Hongyi Yang, Bin Chen, Zhao Liu ("authors") has been accepted for publication by JMIR Publications ("publisher"). A signed copy of this form must be on file with JMIR Publications before the manuscript can be published.

Manuscript #: **59195**
DOI: **10.2196/59195**

Corresponding author: **Zhao Liu**

All authors must sign the agreement. One author must be designated as the correspondent and their name, corresponding address, telephone number, and the title and number of the manuscript must be included within this form. This agreement is made between the authors and JMIR Publications (130 Queens Quay East, Suite 1100, Toronto, ON, M5A 0P6, Canada) for good and sufficient consideration, the receipt of which is acknowledged.

1. Warranties, Representations, and License to Publish

The Authors jointly and severally represent and warrant as a continuing representation and warranty as follows:

1.1 I/we confirm that this article is original and has not been, in whole or in part, formally published in any other peer-reviewed journal, and is not under consideration for publication at another journal. None of the Authors has sold, licensed, assigned, or otherwise encumbered and will not sell, license, assign or otherwise encumber any of the rights granted herein to the Publisher.

1.2 I/we confirm that I am/we are the sole author(s) of the article and have full authority to enter into this agreement and in granting rights to JMIR Publications are not in breach of any other obligation to any other party.

1.3 I/we confirm that the article does not (i) violate any other agreement to which any of the Authors is a party; (ii) include or disclose any

information or data given to the Authors or any of them on the understanding that it would not be published; or (iii) include any information or data that has been obtained or used in the article unlawfully.

1.4 The article does not, and if published will not, infringe upon any copyright, intellectual property rights or proprietary rights of others.

1.5 The article contains nothing unlawful, obscene, defamatory or libelous, or anything which would, if published, constitute a breach of contract,confidence, or confidentiality.

1.6 I/we confirm that I/we have taken due care to ensure the integrity of the article. To my/our—and currently accepted scientific—knowledge, all statements contained in it purporting to be facts are true and any formula or instruction contained in the article will not, if followed accurately, cause any injury, illness, or damage to the user.

1.7 I/we confirm that I/we retain copyright.

1.7.1 I/we confirm that all NIH-affiliated authors have also signed the *NIH Publishing Agreement & Manuscript Cover Sheet* which can be found at https://www.techtransfer.nih.gov/sites/default/files/documents/internal/NIH-Publishing-Agreement-N-Manuscript-Cover-Sheet.pdf and confirm that any provisions therein take precedence over similar provisions in this Agreement, particularly with regard to copyright.

1.8 I/we grant JMIR Publications and third parties, in advance and in perpetuity, the right to use, reproduce or disseminate the published article in its entirety or in part, in any format or medium under a Creative Commons Attribution License (CC-BY 4.0 http://creativecommons.org/licenses/by/4.0/). For greater clarity, this also implies that authors can reproduce the published article or portions of it (e.g. a figure) in a thesis or a book chapter, provided that JMIR Publications is duly identified as the original publisher, that proper attribution of authorship and correct citation details are given (including JMIR journal name and the URL to the original article on the JMIR or sister journals' websites), that a clear notice clarifying the license terms is provided (e.g., a notice saying that the reproduced copy can be freely shared under a Creative Commons Attribution License), that the bibliographic details are not changed, and, if the work is reproduced or disseminated only in part, this fact is clearly and unequivocally indicated.

1.9 I/we grant to JMIR Publications and its successors and assigns, an irrevocable royalty-free world-wide license for the full term of copyright in the all versions of the article (including the submitted, accepted and copyedited manuscripts) to publish it in any language, format or medium and identify JMIR Publications as the original publisher of the journal or medium in which the article first appeared.

1.9.1 I/we grant JMIR Publications the right to edit the article in accordance with the most up-to-date edition of the AMA Manual of Style and JMIR Publications' in-house style guide before publication.

1.10 JMIR Publications has no obligation to publish the article. Examples for scenarios that may arise between acceptance and publication which may lead to a delay or abort production include for example evidence of scientific misconduct, plagiarism, presence of large portions of AI-generated material that was not disclosed on submission, or author disputes. In the event that the article is not published within 18 months of the date of this agreement, I/we acknowledge that these terms and conditions shall cease to apply and neither I/we nor the publisher shall have any further obligations towards the other in respect of the article or these terms and conditions, except for potentially outstanding article processing fees.

1.11 If either the Publisher or any of the Authors becomes aware of an act of copyright infringement of the Work (for example, reproduction of the article by third parties that violate the terms of CC-BY, e.g. not citing the source properly or omitting the CC-BY license from the reproduced work), that party shall immediately notify the other in writing. Either party shall have the right, but not the obligation, to bring an action based on such claims in accordance with applicable law. If the parties proceed jointly, the expenses and recoveries, if any, shall be shared equally. If the parties do not proceed jointly, either party shall have the right to proceed and shall bear the full cost, and retain the full recovery, in the proceedings. If the Publisher proceeds either alone, or jointly with any of the Authors or all the Authors, all the Authors shall permit the action to be brought in the Authors' names and shall take all steps necessary to assist in the prosecution of the action, including attending at all necessary meetings, discoveries, and trial, the execution of such documents as may be required, and cooperating in all reasonable ways to enable the Publisher to proceed. Neither party shall be liable to the other for failing to bring an action or proceeding in respect of copyright infringement.

1.12 The Authors shall jointly and severally indemnify and hold harmless the Publisher and any other party to whom the Publisher or its licensees may extend the representations and warranties contained herein in connection with the publication of the Work or the exercise of

any rights therein or derived therefrom, against any loss, damage, amounts paid in settlement and expenses (including reasonable legal fees and expenses) (collectively, “Losses”) regarding or in connection with any claim, action or proceeding arising from the breach or alleged breach of any of the Authors’ representations, warranties or covenants under this Agreement, according to the following terms:

1.12.1 The Authors or Publisher shall promptly notify the other party in writing of any claim, threat or demand, whether merely asserted or in the form of legal proceedings, made in connection with the Work (the “Claim”) of which either party becomes aware, giving the fullest information obtainable at the time. The Authors and the Publisher shall co-operate fully with each other in defence of any Claim and the Authors’ failure to co-operate in the defence of a Claim shall be deemed a breach of this Agreement;

1.12.2 The Publisher shall defend any Claim made against it with counsel who shall be chosen by the Publisher, at its sole discretion, after consultation with the Authors;

1.12.3 All expenses, including counsel fees, disbursements and all other legal costs, in defending a Claim made against the Publisher (the “Costs”) shall be shared equally by the Authors on the one hand and the Publisher on the other until a final determination of the Claim; the Authors may join in the defence of any Claim with counsel of the Authors’ own choice and at the Authors’ expense;

1.12.4 Where a Claim against the Publisher is successfully defended, the Authors’ indemnity shall be limited to one half of all Costs;

1.12.5 The Publisher may, in its sole discretion, settle any Claim made against the Publisher but only after consultation with the Authors; the Authors and Publisher shall agree on the proportionate shares each shall bear for all costs relating to such settlement; failing such agreement, the Publisher may pursue any remedies available to it based on a breach of the Authors’ obligations including the Authors’ representations and warranties in this Agreement.

1.13 I/we confirm that whenever there is more than one author hereunder, all authors will be jointly and severally responsible for all duties, obligations, representations, warranties and covenants under this Agreement. It is agreed that the Designate (Corresponding) Author shall represent all such joint authors in all matters relating to, or arising out of, this Agreement, and shall have the power to bind all joint authors in respect of any such matter. Further, the Publisher may rely

and act upon any instructions, representation or consents made or given by the Designate to the Publisher without first having to obtain confirmation as to any such instructions, representations or consents from the other joint authors.

2. Letter of Responsibility

2.1. I/we have participated in the conception and design of this work and in the writing of the manuscript and take public responsibility for it. I/we have reviewed the final version of the manuscript and approve it for publication. I/we attest to the validity and legitimacy of the data in the manuscript and agree to be named as author of the manuscript.

3. Disclosure of Funding and Competing Interests

A description of sources of funding and the role of sponsors must be included in the Acknowledgments section of the manuscript. This description should include the involvement, if any, in the following items (in relation to the study):

- The development and production of goods and services
- Review and approval of the manuscript for publication

In addition, authors must disclose in a Conflict of Interest ("COI") section if they have personal or financial interests related to the subject matters discussed in the manuscript. Additional information on Conflicts of Interest can be found at https://support.jmir.org/hc/en-us/articles/115001252671.

If any author indicates that they have a conflict of interest, it is the responsibility of that author to ensure that appropriate language to disclose the COI is added to the final manuscript version (e.g., during copyediting or in the proofs, via the corresponding author or directly in communication with the production office); it is the responsibility of the corresponding author to share the accepted manuscript and final galleys with all authors highlighting the importance of the COI section, and it is the responsibility of the coauthor to check the final galleys to ensure that the COI is accurately and completely disclosed.

If there are no conflicts brought to the attention of the publisher, JMIR Publications will print "None declared."

3.1 I/we certify that financial and material support for this research and work are completely disclosed in the Acknowledgements section.

3.2 I/we warrant that I/we have no further financial interests in the drugs, devices, software, computer programs, internet companies, internet service providers, or procedures described in the enclosed manuscript, *except those disclosed in the Conflict of Interest section of the manuscript*. The Conflict of Interest section also contains all my affiliations and/or financial involvements (e.g., employment,

consultancies, honoraria, stock ownership or options, expert testimony, grants or patents received or pending, royalties) with any organization or entity with a financial interest in or in financial competition with the subject matter or materials discussed in the manuscript.

4. General Terms

4.1 This Agreement shall enure to the benefit of and be binding upon the heirs, executors, administrators and assigns of the Authors and upon the successors and assigns of the Publisher. This agreement shall be interpreted under the laws of the Province of Ontario and the laws of Canada applicable therein and will be treated in all respects as an Ontario contract. The parties attorn to the exclusive jurisdiction of the courts of Ontario. This Agreement contains the entire understanding of the parties with reference to the article. No waiver or modification of any of the terms shall be valid unless made in writing and signed by both the authors and the publisher. No waiver of any breach shall be deemed a waiver of any subsequent breach. Each of the parties will promptly make, do, execute or deliver or cause to be made, done, executed or delivered, all such further acts or documents as the other party may reasonably require from time to time for the purpose of giving effect to this Agreement. Time shall be of the essence of this Agreement.

Do you have a conflict of interest? If you do not please input ‘N/A’.

N/A

Qin Yang	11 / 05 / 2024	Qin Yang
Name of Author	Date	Signature

Do you have a conflict of interest? If you do not please input ‘N/A’.

N/A

Liuxin Zhang	11 / 06 / 2024	Zhang liuxin
Name of Author	Date	Signature

Do you have a conflict of interest? If you do not please input ‘N/A’.

N/A

Fangyuan Chang	11 / 04 / 2024	fangyuan chang

Doc ID: a861ea9c9e5f10d5f76f002afe3945c3868b129d

Name of Author | Date | Signature

Do you have a conflict of interest? If you do not please input ‘N/A’.

N/A

Hongyi Yang | 11 / 06 / 2024 | [illegible]

Name of Author | Date | Signature

Do you have a conflict of interest? If you do not please input ‘N/A’.

N/A

Bin Chen | 11 / 06 / 2024 | Bin Chen

Name of Author | Date | Signature

Do you have a conflict of interest? If you do not please input ‘N/A’.

N

Zhao Liu	11 / 06 / 2024	Zhao Liu
Name of Author	Date	Signature

Doc ID: a861ea9c9e5f10d5f76f002afe3945c3868b129d

Dropbox Sign

Audit trail

**Title**	#59195 - Virtual Reality Interventions for Older Adults with......
**File name**	output.pdf
**Document ID**	a861ea9c9e5f10d5f76f002afe3945c3868b129d
**Audit trail date format**	MM / DD / YYYY
**Status**	● Signed

Document History

SENT	**11 / 04 / 2024**<br>15:09:42 UTC	Sent for signature to Qin Yang (20yangqin@sjtu.edu.cn), Liuxin Zhang (fountain@sjtu.edu.cn), Fangyuan Chang (fangyuanchang@sjtu.edu.cn), Hongyi Yang (agoni97@sjtu.edu.cn), Bin Chen (cb5183298@sjtu.edu.cn) and Zhao Liu (hotlz@sjtu.edu.cn) from admin@jmir.org<br>IP: 3.131.114.152
VIEWED	**11 / 04 / 2024**<br>15:31:38 UTC	Viewed by Fangyuan Chang (fangyuanchang@sjtu.edu.cn)<br>IP: 114.84.121.13
SIGNED	**11 / 04 / 2024**<br>15:33:31 UTC	Signed by Fangyuan Chang (fangyuanchang@sjtu.edu.cn)<br>IP: 114.84.121.13
VIEWED	**11 / 05 / 2024**<br>01:31:12 UTC	Viewed by Qin Yang (20yangqin@sjtu.edu.cn)<br>IP: 103.235.17.57
SIGNED	**11 / 05 / 2024**<br>05:46:07 UTC	Signed by Qin Yang (20yangqin@sjtu.edu.cn)<br>IP: 58.247.22.53

Dropbox Sign

Audit trail

**Title**	#59195 - Virtual Reality Interventions for Older Adults with......
**File name**	output.pdf
**Document ID**	a861ea9c9e5f10d5f76f002afe3945c3868b129d
**Audit trail date format**	MM / DD / YYYY
**Status**	● Signed

Document History

VIEWED	**11 / 06 / 2024**<br>05:59:10 UTC	Viewed by Hongyi Yang (agoni97@sjtu.edu.cn)<br>IP: 146.70.117.231
SIGNED	**11 / 06 / 2024**<br>06:02:04 UTC	Signed by Hongyi Yang (agoni97@sjtu.edu.cn)<br>IP: 146.70.117.231
VIEWED	**11 / 06 / 2024**<br>09:30:58 UTC	Viewed by Zhao Liu (hotlz@sjtu.edu.cn)<br>IP: 138.199.22.66
SIGNED	**11 / 06 / 2024**<br>09:31:46 UTC	Signed by Zhao Liu (hotlz@sjtu.edu.cn)<br>IP: 138.199.22.66
VIEWED	**11 / 06 / 2024**<br>09:36:57 UTC	Viewed by Bin Chen (cb5183298@sjtu.edu.cn)<br>IP: 117.143.47.42
SIGNED	**11 / 06 / 2024**<br>09:37:50 UTC	Signed by Bin Chen (cb5183298@sjtu.edu.cn)<br>IP: 117.143.47.42

**Title**	#59195 - Virtual Reality Interventions for Older Adults with......
**File name**	output.pdf
**Document ID**	a861ea9c9e5f10d5f76f002afe3945c3868b129d
**Audit trail date format**	MM / DD / YYYY
**Status**	● Signed

Document History

VIEWED	**11 / 06 / 2024**<br>10:59:40 UTC	Viewed by Liuxin Zhang (fountain@sjtu.edu.cn)<br>IP: 111.186.5.66
SIGNED	**11 / 06 / 2024**<br>11:06:58 UTC	Signed by Liuxin Zhang (fountain@sjtu.edu.cn)<br>IP: 31.223.184.188
COMPLETED	**11 / 06 / 2024**<br>11:06:58 UTC	The document has been completed.
