# Supplementary material for: Virtual Reality Interventions for Older Adults With Mild Cognitive Impairment: Systematic Review and Meta-Analysis of Randomized Controlled Trials
Source: J Med Internet Res. 2025 Jan 10;27:e59195. doi: 10.2196/59195 (PMC11759915; doi:10.2196/59195)
Supplement: Multimedia Appendix 3 [file jmir_v27i1e59195_app3.pdf]

Appendix 3: Summary of findings and Grading of Recommendations, Assessment, Development, and Evaluations ratings for the main comparisons

Table S1 Summary of findings and Grading of Recommendations, Assessment, Development, and Evaluations ratings for the main comparisons.

**Summary of Key Comparison Findings and Recommendations Grading, Assessing, Developing, and Evaluating Ratings**					
**Outcomes**	**Number of participants (studies)**	**Quality of the evidence[a] (GRADE)[b]**	**Comments**	**Illustrative comparative risks (95% CI)**	
				**Assumed risk control**	**Corresponding risk intervention (95% CI)**
General Cognitive Function	597(13)	⊕⊕⊕⊕⊖ Moderate [c]	Limitation (-1)	No risk assumed	General cognitive function improved in the VR intervention group by 0.09 (-0.07, 0.25)
Performance and Memory	465 (10)	⊕⊕⊕⊕⊖ Moderate [c]	Limitation (-1)	No risk assumed	Performance and memory improved in the VR intervention group by 0.20 (0.02, 0.38)
Attention and Information Processing Speed	410 (9)	⊕⊕⊕⊕⊖ Moderate [c]	Limitation (-1)	No risk assumed	Attention and information processing speed increased in the VR intervention group by 0.25 (0.06, 0.45)
Execution Functions	382(9)	⊕⊕⊕⊕⊖ Moderate [c]	Limitation (-1)	No risk assumed	Executive function improved in the VR intervention group by 0.21 (0.01, 0.41)
Language Proficiency	95(3)	⊕⊖⊖⊖⊖⊖ Very Low [cde]	Limitation (-1) imprecision (-1) Unknown consistency (-1)	No risk assumed	The VR intervention group improved their a) language proficiency by 0.21 (-0.20, 0.61)
Visuospatial Abilities	194(4)	⊕⊕⊕⊖⊖ low [cd]	Limitation (-1) Unknown consistency (-1)	No risk assumed	The VR intervention group improved visuospatial abilities by 0.24 (-0.09, 0.57)
Depression	123(3)	⊕⊕⊕⊖⊖ Low [ce]	Limitation (-1) imprecision (-1)	No risk assumed	Depression decreased in the VR intervention group by 0.06 (-0.29, 0.42)
The Daily Mobility of Individuals	143(4)	⊕⊕⊕⊖⊖ Low [ce]	Limitation (-1) imprecision (-1)	No risk assumed	The VR intervention group improved their ability to perform activities of daily living by 0.10 (-0.23, 0.43)

Gait and Balance	317(7)	⊕⊕⊕⊕⊖ Moderate [c]	Limitation (-1)	No risk assumed	Gait and balance improved in the VR intervention group by 0.05 (-0.17, 0.27)
Muscle Performance	205(4)	⊕⊕⊕⊕⊖ Moderate [c]	Limitation (-1)	No risk assumed	Muscle performance improved in the VR intervention group by 0.19 (-0.09, 0.46)
**Subgroup outcome**					
**With Therapist vs. Without Therapist**					
General Cognitive Function (with therapist)	269(7)	⊕⊕⊕⊕⊖ Moderate [c]	Limitation (-1)	No risk assumed	The VR intervention group with a therapist improved general cognitive function by 0.06 (-0.18, 0.30)
General Cognitive Function (without therapist)	267(5)	⊕⊕⊕⊕⊖ Moderate [c]	Limitation (-1)	No risk assumed	The VR intervention group without a therapist improved general cognitive function by 0.12 (-0.12, 0.36)
Performance and Memory (with therapist)	262(6)	⊕⊕⊕⊖⊖ Low [cd]	Limitation (-1) Unknown consistency (-1)	No risk assumed	Performance and memory improved by 0.17 (-0.07, 0.41) in the VR intervention group with a therapist
Performance and Memory (without therapist)	118(2)	⊕⊕⊕⊖⊖ Low [cd]	Limitation (-1) Unknown consistency (-1)	No risk assumed	Performance and memory improved by 0.39 (0.03, 0.76) in the VR intervention group without a therapist
Attention and Information Processing Speed (with therapist)	193(5)	⊕⊕⊕⊖⊖ Low [cd]	Limitation (-1) Unknown consistency (-1)	No risk assumed	The VR intervention group with a therapist improved attention and information processing speed by 0.14 (-0.14, 0.43)
Attention and Information Processing Speed (without therapist)	132(2)	⊕⊕⊕⊖⊖ Low [cd]	Limitation (-1) Unknown consistency (-1)	No risk assumed	The VR intervention group without a therapist improved attention and information processing speed by 0.39 (0.03, 0.75)
Executive Function (with therapist)	198(6)	⊕⊕⊕⊕⊖ Moderate [c]	Limitation (-1)	No risk assumed	The VR intervention group with a therapist improved executive function by 0.20 (-0.08, 0.48)
Executive Function (without therapist)	184(3)	⊕⊕⊕⊕⊖ Moderate [c]	Limitation (-1)	No risk assumed	The VR intervention group without a therapist improved executive function by 0.25 (-0.04, 0.54)
**Immersion level: immersive vs. semi-immersive vs. non-immersive vs. immersive and semi-immersive**					
Attention and Information Processing Speed	255(5)	⊕⊕⊕⊖⊖ Low [cd]	Limitation (-1) Unknown consistency (-1)	No risk assumed	The immersive VR intervention group increased attention and information processing speed by

(Immersive)					0.25 (0.01, 0.50)
Attention and Information Processing Speed (Semi-Immersive)	35(1)	⊕⊖⊖⊖ Very low [cde]	Limitation (-1) imprecision (-1) Unknown consistency (-1)	No risk assumed	The semi-immersive VR intervention group improved attention and information processing speed by 0.09 (-0.57, 0.76)
Attention and information processing speed (non-immersive)	96(2)	⊕⊖⊖⊖ Very low [cde]	Limitation (-1) imprecision (-1) Unknown consistency (-1)	No risk assumed	The non-immersive VR intervention group improved attention and information processing speed by 0.29 (-0.11, 0.69)
**Interventions: VR cognitive training VS VR physical training VS VR cognitive-motor dual-task training VS VR nostalgia therapy**					
General Cognitive Function (VR Cognitive Training)	261(5)	⊕⊕⊕⊖⊖ Low [cd]	Limitation (-1) Unknown consistency (-1)	No risk assumed	The VR cognitive training intervention improved general cognitive function by 0.02 (-0.22, 0.26)
General Cognitive Function (VR Body Training)	120(2)	⊕⊕⊕⊖⊖ Low [cd]	Limitation (-1) Unknown consistency (-1)	No risk assumed	The VR physical training intervention improved general cognitive function by 0.07 (-0.29, 0.43)
General Cognitive Function (VR Cognitive Motor Dual Task Training)	216(6)	⊕⊕⊕⊖⊖ Low [cd]	Limitation (-1) Unknown consistency (-1)	No risk assumed	The VR cognitive-motor dual-task training intervention improved general cognitive function by 0.17 (-0.09, 0.44)
Attention and Information Processing Speed (VR Cognitive Training)	232(5)	⊕⊕⊕⊕⊖ Moderate [c]	Limitation (-1)	No risk assumed	VR cognitive training intervention attention and information processing speed increased by 0.32 (0.06, 0.58)
Attention and Information Processing Speed (VR Body Training)	60(1)	⊕⊖⊖⊖ Very low [cde]	Limitation (-1) imprecision (-1) Unknown consistency (-1)	No risk assumed	VR physical training intervention attention and information processing speed increased by 0.06 (-0.45, 0.57)
Attention and Information Processing Speed (VR Cognitive Motor Dual Task Training)	90(2)	⊕⊖⊖⊖ Very low [cde]	Limitation (-1) imprecision (-1) Unknown consistency (-1)	No risk assumed	VR Cognitive-Motor Dual-Task Training Intervention Attention and Information Processing Speed Increased by 0.21 (-0.19, 0.61)
Attention and Information Processing Speed (VR Program)	24(1)	⊕⊖⊖⊖ Very low [cde]	Limitation (-1) imprecision (-1) Unknown consistency (-1)	No risk assumed	VR programs interfered with attention and information processing speed by 0.30 (-0.50, 1.11)

[a]GRADE Working Group grades of evidence: High quality: further research is very unlikely to change our confidence in the estimate of effect; Moderate High quality: further research is very unlikely to change our confidence in the estimate of effect; Moderate quality: further research is likely to have an important impact on our confidence in the estimate of effect and may change the estimate; Low quality. further research is very likely to have an important impact on our confidence in the estimate of effect and is likely to change the estimate; Very low Low quality: further research is very likely to have an important impact on our confidence in the estimate of effect and is likely to change the estimate; Very low quality: we are very uncertain about the estimate.

[b] GRADE: Grading of Recommendations, Assessment, Development, and Evaluations.

[c] Majority of the evidence comes from studies with unclear randomization and/or allocation concealment.

[d] Unknown consistency and/or publication bias.

[e] Total sample size is small. Total effect size has CIs crossing the no effect line.
