# Supplementary material for: Virtual Reality Interventions for Older Adults With Mild Cognitive Impairment: Systematic Review and Meta-Analysis of Randomized Controlled Trials
Source: J Med Internet Res. 2025 Jan 10;27:e59195. doi: 10.2196/59195 (PMC11759915; doi:10.2196/59195)
Supplement: Multimedia Appendix 4 [file jmir_v27i1e59195_app4.pdf]

Appendix 4: Forest Plots of Secondary Outcomes

Contents:

Other cognitive-related abilities *1*
Depression *1*
The Daily Mobility of Individuals *2*
Physical fitnesse *2*

Other cognitive-related abilities

Study or Subgroup	Experimental Mean	Experimental SD	Experimental Total	Control Mean	Control SD	Control Total	Weight	Std. Mean Difference IV, Random, 95% CI
05 Park et al 2020	1.9	5.61	10	0.36	4.41	11	22.1%	0.29 [-0.57, 1.16]
08 Manenti et al 2020	1.3	26.49	18	-1.8	26.09	14	33.7%	0.11 [-0.58, 0.81]
14 Kang et al 2021	0.42	0.94	23	0.14	1.38	19	44.2%	0.24 [-0.37, 0.85]
**Total (95% CI)**			**51**			**44**	**100.0%**	**0.21 [-0.20, 0.61]**

Heterogeneity: Tau² = 0.00; Chi² = 0.12, df = 2 (P = 0.94); I² = 0%
Test for overall effect: Z = 1.01 (P = 0.31)

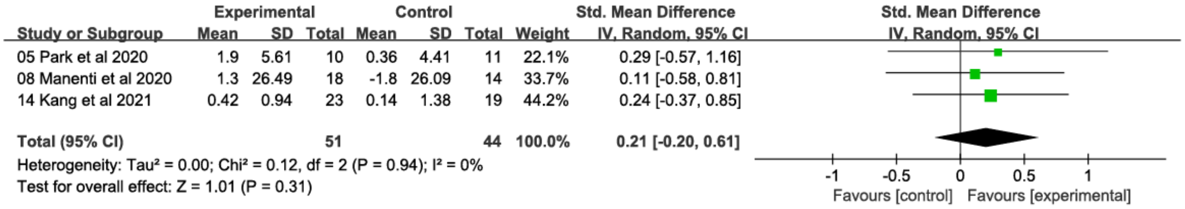

Figure S1: Forest plot of language proficiency: comparison of Language Proficiency at post-intervention time points based on VR intervention versus conventional treatment or no intervention control group.

Study or Subgroup	Experimental Mean	Experimental SD	Experimental Total	Control Mean	Control SD	Control Total	Weight	Std. Mean Difference IV, Random, 95% CI
08 Manenti et al 2020	0.3	1.47	18	0.1	1.57	17	19.9%	0.13 [-0.54, 0.79]
14 Kang et al 2021	0.53	0.7	23	-0.4	1.51	19	21.4%	0.80 [0.17, 1.44]
16 Park et al 2022	2	25.82	28	0.04	24	28	28.5%	0.08 [-0.45, 0.60]
18 Torpil et al 2017	1.25	15.1	30	0.36	14.94	31	30.3%	0.06 [-0.44, 0.56]
**Total (95% CI)**			**99**			**95**	**100.0%**	**0.24 [-0.09, 0.57]**

Heterogeneity: Tau² = 0.03; Chi² = 3.99, df = 3 (P = 0.26); I² = 25%
Test for overall effect: Z = 1.41 (P = 0.16)

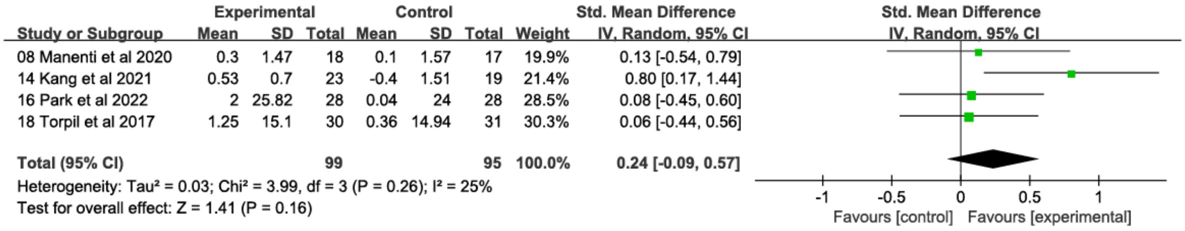

Figure S2: Forest plot of visuospatial abilities: comparison of visuospatial abilities at post-intervention time points based on VR intervention versus conventional treatment or no intervention control group.

Depression

Study or Subgroup	Experimental Mean	Experimental SD	Experimental Total	Control Mean	Control SD	Control Total	Weight	Std. Mean Difference IV, Random, 95% CI
05 Park et al 2020	1.1	5.08	10	0.55	5.32	11	17.1%	0.10 [-0.76, 0.96]
14 Kang et al 2021	1.74	11.48	23	0.45	10.24	19	33.9%	0.12 [-0.49, 0.72]
15 Hsieh et al 2018	-0.52	2.6	31	-0.56	2.57	29	49.0%	0.02 [-0.49, 0.52]
**Total (95% CI)**			**64**			**59**	**100.0%**	**0.06 [-0.29, 0.42]**

Heterogeneity: Tau² = 0.00; Chi² = 0.07, df = 2 (P = 0.97); I² = 0%
Test for overall effect: Z = 0.35 (P = 0.72)

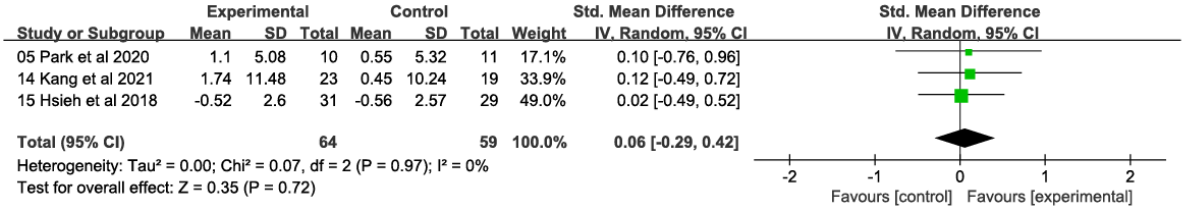

Figure S3 Forest plot of depression: comparison of depression at post-intervention time points based on VR intervention versus conventional treatment or no intervention control group.

The Daily Mobility of Individuals

Study or Subgroup	Experimental Mean	Experimental SD	Experimental Total	Control Mean	Control SD	Control Total	Weight	Std. Mean Difference IV, Random, 95% CI
01 Park et al 2022	2.94	18.12	16	-1.57	16.81	16	22.3%	0.25 [-0.44, 0.95]
06 Liao et al 2020	1.61	18.8	18	0.44	17.32	16	23.8%	0.06 [-0.61, 0.74]
08 Manenti et al 2020	-0.1	32.4	18	0.2	33.53	17	24.6%	-0.01 [-0.67, 0.65]
14 Kang et al 2021	1.22	30.09	23	-2.22	29.99	19	29.2%	0.11 [-0.50, 0.72]
**Total (95% CI)**			**75**			**68**	**100.0%**	**0.10 [-0.23, 0.43]**

Heterogeneity: Tau² = 0.00; Chi² = 0.30, df = 3 (P = 0.96); I² = 0%
Test for overall effect: Z = 0.61 (P = 0.54)

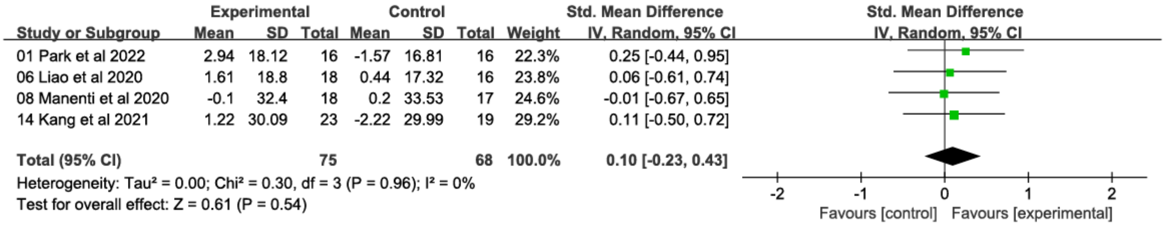

Figure S4 Forest plot of the daily mobility of individuals: comparison of the daily mobility of individuals at post-intervention time points based on VR intervention versus conventional treatment or no intervention control group.

Physical fitness

Study or Subgroup	Experimental Mean	Experimental SD	Experimental Total	Control Mean	Control SD	Control Total	Weight	Std. Mean Difference IV, Random, 95% CI
04 Thapa et al 2020	0.04	1.05	34	-0.06	1.02	34	21.5%	0.10 [-0.38, 0.57]
09 Kwan et al 2021	0.35	9.15	9	4.7	9.38	8	5.2%	-0.45 [-1.41, 0.52]
10 Delbroek et al 2017	1.4	13.74	8	2	17.41	9	5.4%	-0.04 [-0.99, 0.92]
11 Choi et al 2019	1.35	8.1	30	0.3	9.38	30	19.0%	0.12 [-0.39, 0.62]
12 Liao et al 2019	14.4	72.24	18	5.3	67.89	16	10.7%	0.13 [-0.55, 0.80]
15 Hsieh et al 2018	44.41	170	31	35.43	229.6	29	19.0%	0.04 [-0.46, 0.55]
17 Hwang et al 2022	1.25	15.1	30	0.36	14.94	31	19.3%	0.06 [-0.44, 0.56]
**Total (95% CI)**			**160**			**157**	**100.0%**	**0.05 [-0.17, 0.27]**

Heterogeneity: Tau² = 0.00; Chi² = 1.20, df = 6 (P = 0.98); I² = 0%
Test for overall effect: Z = 0.45 (P = 0.65)

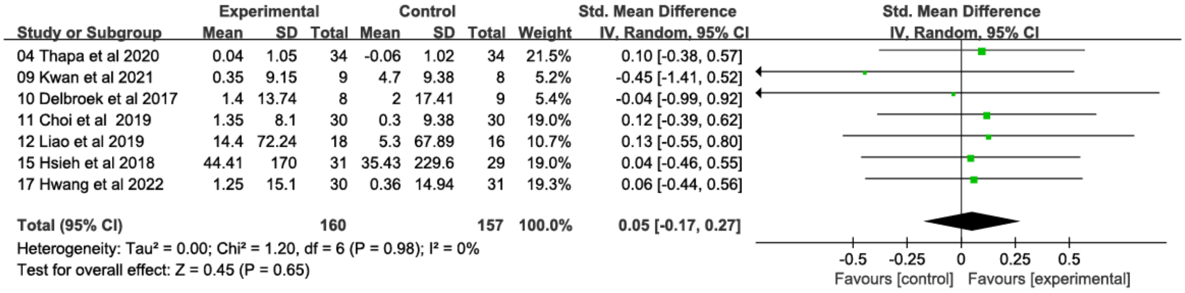

Figure S5 Forest plot of gait and balance: comparison of gait and balance at post-intervention time points based on VR intervention versus conventional treatment or no intervention control group.

Study or Subgroup	Experimental Mean	Experimental SD	Experimental Total	Control Mean	Control SD	Control Total	Weight	Std. Mean Difference IV, Random, 95% CI
04 Thapa et al 2020	2.2	22.23	34	0.5	21.62	34	33.4%	0.08 [-0.40, 0.55]
09 Kwan et al 2021	1	13.96	9	1.3	13.44	8	8.3%	-0.02 [-0.97, 0.93]
11 Choi et al 2019	6.35	25.35	30	0.64	21	30	29.2%	0.24 [-0.27, 0.75]
15 Hsieh et al 2018	0.34	8.04	31	-2.2	7.85	29	29.1%	0.32 [-0.19, 0.83]
**Total (95% CI)**			**104**			**101**	**100.0%**	**0.19 [-0.09, 0.46]**

Heterogeneity: Tau² = 0.00; Chi² = 0.68, df = 3 (P = 0.88); I² = 0%
Test for overall effect: Z = 1.33 (P = 0.18)

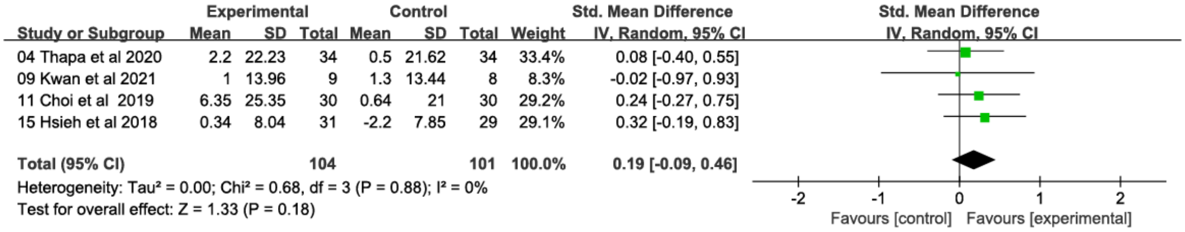

Figure S6 Forest plot of muscle performance: comparison of muscle performance at post-intervention time points based on VR intervention versus conventional treatment or no intervention control group.
