# Supplementary material for: Virtual Reality Interventions for Older Adults With Mild Cognitive Impairment: Systematic Review and Meta-Analysis of Randomized Controlled Trials
Source: J Med Internet Res. 2025 Jan 10;27:e59195. doi: 10.2196/59195 (PMC11759915; doi:10.2196/59195)
Supplement: Multimedia Appendix 5 [file jmir_v27i1e59195_app5.pdf]

Appendix 5: Funnel Plots of Review Comparisons

Contents:

General Cognitive Function *1*
Memory and Performance *1*
Attention and Information Processing Speed *2*
Executive Function *2*

General Cognitive Function

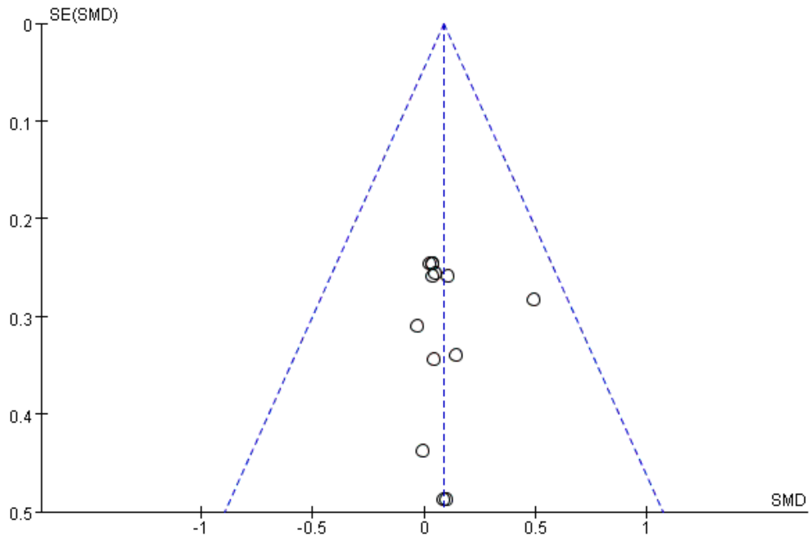

Figure S1: Funnel plot of general cognitive function

Memory and Performance

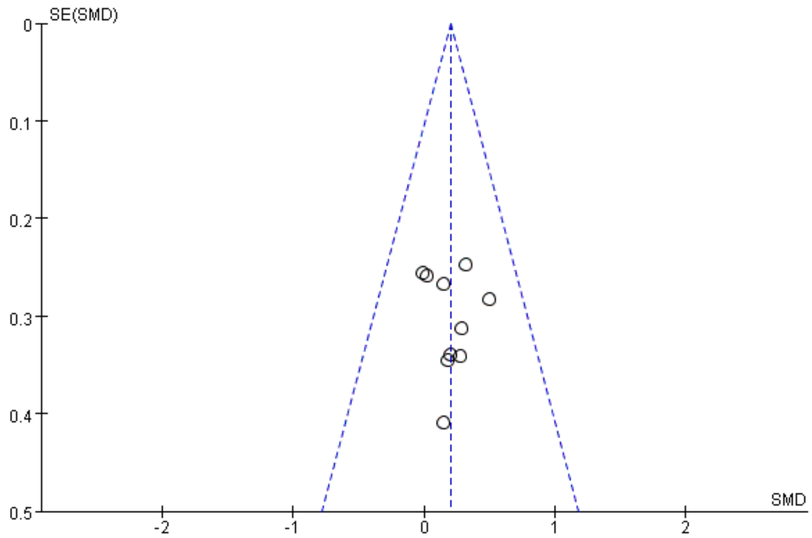

Figure S2 Funnel plot of memory and performance

Attention and Information Processing Speed

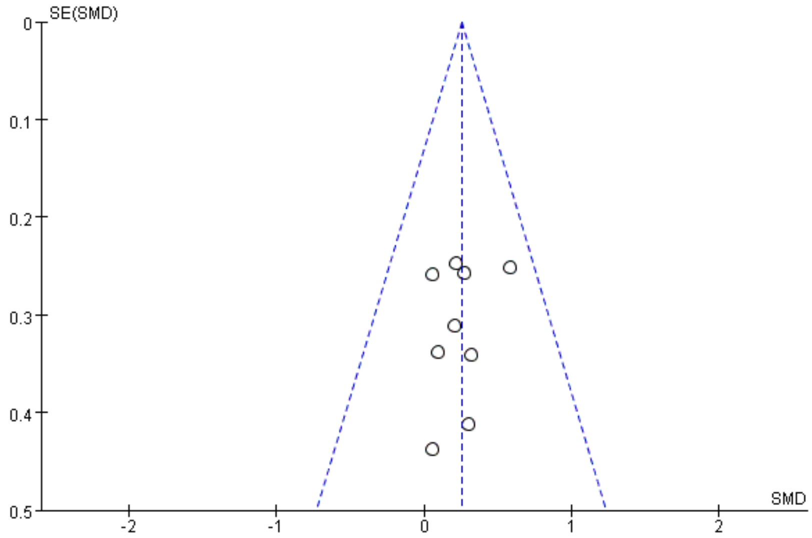

Figure S3 Funnel plot of attention and information processing speed

Executive Function

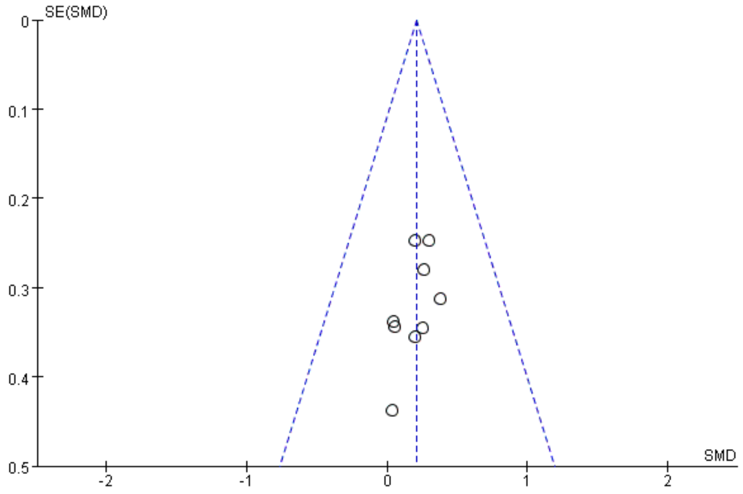

Figure S4 Funnel plot of executive function
