# Supplementary material for: Virtual Reality Interventions for Older Adults With Mild Cognitive Impairment: Systematic Review and Meta-Analysis of Randomized Controlled Trials
Source: J Med Internet Res. 2025 Jan 10;27:e59195. doi: 10.2196/59195 (PMC11759915; doi:10.2196/59195)
Supplement: Multimedia Appendix 6 [file jmir_v27i1e59195_app7.pdf]

PRISMA 2020 Checklist

Section and Topic	Item #	Checklist item	Location where item is reported
**TITLE**			
Title	1	Virtual Reality Interventions for older adults with mild cognitive impairment: a Systematic Review and Meta-analysis of Randomized Controlled Trials	1
**ABSTRACT**			
Abstract	2	**Objective**<br>To analyze the effects of a virtual reality (VR) intervention on the outcomes of older adults with MCI.<br>**Design**<br>Systematic review of randomized controlled trials (RCTs).<br>**Data sources**<br>The Web of Science, Embase, Ovid, and PubMed databases were searched from January 2013 to December 2023.<br>**Eligibility criteria**<br>RCTs that assessed the effects of VR-based intervention tools on MCI.<br>**Results**<br>A total of 18 studies involving 722 older adults with MCI were included in this review. VR was delivered through different immersion levels with VR cognitive training, VR physical training, or VR cognitive-motor dual-task training. Studies reported significant improvements in performance and memory (SMD 0.20; 95% CI 0.02 to 0.38), attention and information processing speed (SMD 0.25; 95% CI 0.06 to 0.45), and executive function (SMD 0.22; 95% CI 0.02 to 0.42) showed significant improvements. VR without therapist involvement showed significant improvements in performance and memory as well as attention and information processing speed; VR cognitive training also resulted in significant improvements in attention and information processing speed in individuals with MCI. Additionally, immersive VR had a significant impact on improving attention, information processing speed, and executive function. The effects of the intervention were very small in terms of general cognitive function, language proficiency, visuospatial abilities, depression, daily living ability, muscle performance, and gait and balance. According to the GRADE approach, the quality of evidence was rated as moderate in the areas of general cognitive function, performance and memory, attention and information processing speed, executive function, and gait and balance, and as low and very low in the areas of verbal ability, visuospatial abilities, depression, ability to perform daily living, and muscle performance.<br>**Conclusions**<br>VR-based interventions may result in improvement in performance and memory, attention and information processing speed, and executive function in older adults with MCI. This improvement is related to the presence or absence of a therapist during the intervention, the content of the VR intervention, and the VR immersion effect. The quality of evidence is moderate to low, and further research is required to confirm the findings of the review and assess the effects of VR technology on other health-related outcomes.	1
**INTRODUCTION**			
Rationale	3	as the worldwide occurrence of Alzheimer's disease (AD) rises, the economic burden on society will also increase significantly, with greater disease severity being linked to higher expenses. Treatments can be implemented to slow down the advancement of dementia during the mild cognitive impairment (MCI) phase. Since there are few medications or dietary therapy that can improve cognitive function or slow MCI progression, non-pharmacological treatments, virtual reality (VR) technology has received attention. Accumulating evidence has demonstrated improvement in cognitive functions (general cognitive ability, memory, attention, and executive function) using VR-based intervention in older adults with MCI. Moreover, some studies have reported that VR-based intervention can effectively improve their non-cognitive abilities (mood, daily living in individuals) in older adults with MCI.<br>Nevertheless, whether VR intervention is feasible remains controversial. The efficacy of digital interventions blended with behavioural science must be thoroughly assessed before clinical implementation/application. In addition, an updated assessment is required due to rapid advances in (VR) technology over the past few years	2
Objectives	4	This review aims to assess the effects of VR technology-based interventions on older adults with MCI.	3
**METHODS**			

PRISMA 2020 Checklist

Section and Topic	Item #	Checklist item	Location where item is reported
Eligibility criteria	5	Studies fulfilling the following criteria were included in the present review and meta-analysis: randomized controlled trials (RCTs); participants included those (older adults ≥55 years of age) with a confirmed diagnosis of MCI by neurologic examination or neuropsychological assessment. Healthy older adults, patients with schizophrenia, depression, Parkinson's, and patients who did not specify the level of cognitive deficits in participants were excluded.; and those addressing at least one component of general cognitive function, memory and performance, attention and information processing speed, executive function, language proficiency, visuospatial abilities, depression, the daily mobility of individuals, gait and balance, or muscle performance. Studies published in any language other than English and those without sufficient data were excluded. Only peer-reviewed studies involving participants ≥ 18 of age were eligible for inclusion.	4
Information sources	6	The literature search was performed using the Web of Science, Embase, Ovid, and PubMed databases, from date of January 2013 to December 2023. In addition, the reference lists of relevant reviews and selected articles were manually examined for potentially relevant/eligible trials.	4
Search strategy	7	(Appendix 1)	
Selection process	8	The initial search retrieved 571 articles after removing duplicates, of which 493 that did not fulfill the inclusion criteria were excluded. Subsequently, 77 eligible full-text articles were reviewed, of which 18 RCTs were ultimately included in the quantitative analysis (Figure 1).	4
Data collection process	9	All duplicate references were removed. Subsequently, titles and abstracts were manually screened for potentially eligible studies by two reviewers (QY and ZL), and relevant RCTs were identified. The full text was then retrieved by both reviewers to assess eligibility for inclusion. Disagreements between the researchers were resolved through discussion or consultation with a third reviewer (FC). The data were cross validated by two researchers (HY and LZ) using EndNote 20 (Clarivate Analytics, Philadelphia, PA, USA).	6
Data items	10a	The dataset comprised study information, participant characteristics, type of intervention, immersion level, attrition at postintervention, and outcome measures, including general cognitive function, memory and performance, attention and information processing speed, executive function, language proficiency, visuospatial abilities, depression, the daily mobility of individuals, gait and balance, and muscle performance. If a study used multiple pain scales, the scale with the highest sensitivity to change(s) was used.	5
	10b	(1) General Cognitive Function: the Brief Mental State Examination (MMSE), the Montreal Cognitive Assessment (MoCA), Korean version of the Modified Mental State Examination-Dementia Screening Test (MMSE-DS), the Cognitive Abilities Screening Instrument (CASI), Subcategory VST of CNT 4.0, Montreal Cognitive Assessment and GP Cognitive Assessment (dementia screening tool) and Loewenstein Occupational Therapy Cognitive Assessment – Aging (LOTCA-G)<br>(2) Memory and Performance: the Digit Span Test (DST-Forward/Backward), Rey Auditory Verbal Learning Test (RAVLT), Wechsler Memory Scale, Third Edition (WMS-III), Multifactorial Memory Questionnaire (MMQ), Chinese Verbal Learning Test (CVVLT), 20-item version of the Everyday Memory Questionnaire (range: 20-180) and Seoul Verbal Learning Test (SVLT).<br>(3) Attention and Information Processing Speed: Trajectory Making Test (TMT) A and B, Symbol Digit Substitution Test (SDST), Attention Matrix Test (AM), and Digit Span Test (DST-Forward/Backward).<br>(4) Executive Function: Korean version of the Executive Function Performance Test (EFPT-K), Symbol Digit Substitution Test (SDST), Trajectory Making Test (TMT)B, Stroop Color and Word Test (SCWT), Executive Interview 25 (EXIT-25), Frontal Assessment Battery (FAB)<br>(1) Language Proficiency: Word Fluency Test (WFT) (category and letter fluency), Verbal Fluency (phonological, FPL; semantic, FPC), Battery for Analysis of Aphasic Deficits (B.A.D.A.), Korean version of the Boston Naming Test (K-BNT)<br>(2) Visuospatial Abilities: Rey-Osterrieth Complex Figure Test (RCFT), Clock Drawing Test (CDT), and Weschsler Adult Intelligence Scale Revised Block Design Test (WAIS-BDT)<br>(3) Depression: 30-item Geriatric Depression Scale (GDS), 15-item Geriatric Depression Scale (GDS-15), and Multidimensional Observation Scale for Elderly Subjects (MOSES)<br>(4) The Daily Mobility of Individuals: the Lawton Instrumental Activities of Daily Living (IADL) scale, the Quality of Life in Alzheimer's Disease (QOL-AD) scale, and the Multidimensional Observation Scale for Elderly Subjects (MOSES)<br>(5) Gait and Balance: Gait Speed Test, Timed Up and Go Test (TUG), Instrumented Timed Up and Go Test (iTUG), LOS values and Six Minute Walk Test (6MWT)<br>(6) Muscle Performance: Hand Gauge Measurement (HGS), Arm Flexion Test, and Lower Body Strength using the 30-second Sit-Stand Test	5

PRISMA 2020 Checklist

Section and Topic	Item #	Checklist item	Location where item is reported
		(30-second STS)	
Study risk of bias assessment	11	Risk of bias was assessed using the Risk-of-Bias Tool in the Cochrane Handbook of Systematic Evaluation. Seven domains, including random sequence generation, allocation concealment, blinding of participants and personnel, incomplete outcome data, selective reporting, and other biases, were used to evaluate quality of evidence. Each domain was assigned a judgement of low, high, or unclear risk of bias.	7
Effect measures	12	a positive SMD value for general cognitive function, memory and performance, attention and information processing speed, executive function, language proficiency, visuospatial abilities, depression, the daily mobility of individuals, gait and balance, and muscle performance favour VR-based intervention. The magnitude of SMD was interpreted in accordance with guidelines reported by Cohen [41], as follows: SMD < 0.2 (small); 0.2 to 0.8 (medium); and > 0.8 (large). For clinical interpretation, the mean differences were calculated.	7
Synthesis methods	13a	13 trials reported general cognitive ability, 10 trials reported memory and performance, 9 trials reported attention and information processing speed, and 9 trials reported executive function. Language proficiency, visuospatial abilities, depression, the daily mobility of individuals, gait and balance, and muscle performance, respectively (Appendix 4).	7
	13b	The authors of studies with missing data were contacted. When the authors were unavailable, data were estimated using recommendations from the Cochrane Handbook (e.g., estimation of SD from standard error [SEs]). In trials in which SD was not reported, missing data were imputed from 95% confidence intervals (CIs), SEs, p-values, baseline changes, graphical representations, medians, and interquartile ranges (IQRs), or SDs from baseline. Trials in which imputations were not possible were excluded from the quantitative analysis.	7
	13c	Standardised mean differences (SMDs) were calculated to standardise the results to a uniform scale when studies assessed the same outcomes using different instruments.	7
	13d	We used meta-analysis to assess outcome effect values. If I2>50%, a random effects model was used. All analyses were performed using Review Manager version 5.3.	7
	13e	Subgroups were defined in terms of the type of intervention content (VR cognitive training, VR physical training, VR cognitive-motor dual-task training, and VR program), type of immersion Level (immersive VR, Semi-immersive VR, non-immersive VR, and combined immersive and non-immersive VR), and the therapist involvement.	5
	13f	Sensitivity and subgroup analyses were performed to assess the potential impact of the sources of heterogeneity. To investigate the potential impact of methodological quality on the estimates, a sensitivity analysis was performed by removing ten study at a time, and trials with poor methodological quality were removed.	5
Reporting bias assessment	14	Quality level in this meta-analysis was evaluated according to the Grading of Recommendations Assessment, Development, and Evaluation (GRADE) approach. Quality of evidence was classified as high, moderate, low, or very low.	7
Certainty assessment	15	Heterogeneity of the pooled studies was examined using the chi-squared test and the I^2 statistic, with $I^2 > 50\%$ indicating substantial heterogeneity. Publication bias was assessed by visual inspection of funnel plots and Egger's test for meta-analyses of ≥ 10 trials. All analyses were performed using Review Manager version 5.3.	7
**RESULTS**			
Study selection	16a	The initial search retrieved 571 articles after removing duplicates, of which 493 that did not fulfill the inclusion criteria were excluded. Subsequently, 77 eligible full-text articles were reviewed, of which 18 RCTs were ultimately included in the quantitative analysis (Figure 1).	8
	16b	0 additional records were found.	8
Study characteristics	17	18 studies were included, with a total of 8 studies conducted in Korea, 2 in Hong Kong, China, 2 in Taipei, China, and 6 in mainland China, Greece, Brazil, France, Turkey, and Japan. The number of participants in the studies ranged from 17 to 68. The median (IQR) age was 73.15 (5.38) years.18 Most participants in the studies were female (median 63.6%, IQR 25.4%). Nine studies reported participants' years of education, with a median (IQR) age of 8.56 (2.50) years. Ten studies reported participants' baseline MMES scores, with a median (IQR) score of 26.28 (1.55).	8

PRISMA 2020 Checklist

Section and Topic	Item #	Checklist item	Location where item is reported
Risk of bias in studies	18	In general, the most frequent risks of bias for RCTs were blinding of participants/personnel (100%) and selection bias (55.6%). Detection bias accounted for the second most frequent risks of bias (50%). The overall confidence in the cumulative evidence varied from very low to moderate, with low confidence being the most commonly identified ((Appendix 3).	10
Results of individual studies	19	(Appendix 2)	9
Results of syntheses	20a	(Figure 3-11 and Appendix 4)	9
	20b	For general cognitive function, 13 trials with 597 patients reported a small but not significant effect of VR-based interventions in improve general cognitive function (SMD 0.09; 95% CI -0.07 to 0.25), with moderate -quality evidence. However, the findings of this review indicate that using VR as an intervention resulted in significant improvements in performance and memory (SMD 0.20; 95% CI 0.02 to 0.38), speed of attention and information processing (SMD 0.25; 95% CI 0.06 to 0.45), and executive function (SMD 0.22; 95% CI 0.02 to 0.42) with moderate -quality evidence. Moreover, the results of subgroup analyses show that the VR intervention without therapist involvement had better results in terms of improving performance and memory as well as in terms of attention and speed of information processing compared to the VR intervention with therapist involvement. The results of the subgroup analysis regarding the content of the VR intervention indicate that VR cognitive training produces significant effects in improving attention and information processing speed compared to VR physical training, VR cognitive-motor dual-task training, and VR programs. Furthermore, immersive VR produced significant effects in terms of improving attention and information processing speed and in terms of executive function compared to semi-immersive VR, non-immersive VR, and combined immersive and semi-immersive VR intervention situations.	11
	20c	We were unable to fully explore the reasons for heterogeneity because many covariates for psychotherapy-based digital tools in MCI were not usually reported in the trials.	17
	20d	Little evidence of funnel plot asymmetry in the intervention effects for general cognitive function, performance and memory, attention and speed of information processing, and executive function (Appendix 4)	17
Reporting biases	21	Summarized according to studies, the most frequent risk of bias for randomized controlled trials were blinding of participants/personnel (100%) and selection bias (55.6%). Similarly, detection bias was also the most frequent risk of bias (50% studies high risk of bias). Overall confidence in cumulative evidence varied from very low to moderate with low confidence most commonly identified.	10
Certainty of evidence	22	(Appendix 3) presented the GRADE evaluation of evidence	10
**DISCUSSION**			
Discussion	23a	This systematic review provides moderate to very low-quality evidence of VR-based interventions in treating older adults with MCI. Our study did not discover any notable disparities in general cognitive function and any secondary outcomes. However, VR-based interventions were proved to be effective on performance and memory, speed of attention and information processing, and executive function with low-quality evidence. However, further high- quality trials should provide more precise estimates.	17
	23b	We were unable to fully explore the reasons for heterogeneity because many covariates for psychotherapy-based digital tool in MCI are normally not recorded in trials.	17
	23c	First, only studies published in English were considered. Second, it is difficult to blind interventions and outcome assessments; as a result, all studies suffer from performance and detection bias. In addition, some of the most robust studies were at high risk of bias, reducing the overall quality of the evidence. Third, the sample sizes of most studies were small, limiting the statistical power and generalizability of the results, and secondary outcomes such as language proficiency, depression, and ability to perform activities of the daily mobility of Individuals were only reported in 4 studies each. Fourth, defining thresholds of dysfunction remains a difficult task, and the included studies differed in inconsistency in their inclusion criteria for participants with MCI. Finally, most studies did not take into account factors that can modulate participants' cognitive functioning, mood, the daily mobility of Individuals, and physical fitness (e.g., cognitive reserve, diet, sleep, and physical and cognitive activities outside of the intervention). However, our findings can still provide compelling insights into the future of VR intervention design for older adults with MCI.	20

PRISMA 2020 Checklist

Section and Topic	Item #	Checklist item	Location where item is reported
	23d	The VR intervention resulted in notable enhancements in performance and memory, attention and information processing speed, and executive function among older adults with MCI. Furthermore, VR training without the presence of a therapist resulted in notable enhancements in performance, memory, attention, and information processing speed. VR cognitive training also led to significant improvements in attention and information processing speed abilities among individuals with MCI. Additionally, immersive VR training had significant effects on improving attention, information processing speed, and executive function. However, more research is needed to directly compare these various training programmes and validate this conclusion. Hence, forthcoming research should take into account the diversity of patient populations, the precise impacts of VR therapies, the length and amounts of interventions, and the accuracy of measuring techniques in order to comprehensively comprehend the potential advantages of VR in patients with MCI.	18
**OTHER INFORMATION**			
Registration and protocol	24a	The present review was performed in accordance with the Preferred Reporting Items for Systematic Reviews and Meta-Analysis (i.e., “PRISMA”) and guidelines published in the Cochrane Handbook of Systematic Evaluation. The study protocol was registered with the International Register of Prospective Systematic Reviews (PROSPERO): CRD42024503488.	3
	24b	The study protocol was registered with the International Register of Prospective Systematic Reviews (PROSPERO)	3
	24c	There is no change to the title, which is “Virtual Reality Interventions for older adults with mild cognitive impairment: a Systematic Review and Meta-analysis of Randomized Controlled Trials”	
Support	25	Non-financial support	21
Competing interests	26	No any competing interests of review authors.	21
Availability of data, code and other materials	27	Specific data can be requested from the authors, if needed: template data collection forms; data extracted from included studies; data used for all analyses; analytic code; any other materials used in the review.	

From: Page MJ, McKenzie JE, Bossuyt PM, Boutron I, Hoffmann TC, Mulrow CD, et al. The PRISMA 2020 statement: an updated guideline for reporting systematic reviews. BMJ 2021;372:n71. doi: 10.1136/bmj.n71

For more information, visit: http://www.prisma-statement.org/
